# Dihomooxacalix[4]arene-Based Fluorescent Receptors for Anion and Organic Ion Pair Recognition

**DOI:** 10.3390/molecules25204708

**Published:** 2020-10-14

**Authors:** Alexandre S. Miranda, Paula M. Marcos, José R. Ascenso, Mário N. Berberan-Santos, Rachel Schurhammer, Neal Hickey, Silvano Geremia

**Affiliations:** 1 Centro de Química Estrutural, Faculdade de Ciências da Universidade de Lisboa, Edifício C8, 1749-016 Lisboa, Portugal; miranda.m.alexandre@gmail.com; 2 IBB–Institute for Bioengineering and Biosciences, Instituto Superior Técnico, Universidade de Lisboa, 1049-001 Lisboa, Portugal; berberan@tecnico.ulisboa.pt; 3 Faculdade de Farmácia da Universidade de Lisboa, Av. Prof. Gama Pinto, 1649-003 Lisboa, Portugal; 4 Instituto Superior Técnico, CQE, Complexo I, Av. Rovisco Pais, 1049-001 Lisboa, Portugal; jose.ascenso@ist.utl.pt; 5 Laboratoire de Modélisation et Simulations Moléculaires, Université de Strasbourg, UMR 7140, F-67000 Strasbourg, France; rschurhammer@unistra.fr; 6 Department of Chemical and Pharmaceutical Sciences, Centre of Excellence in Biocrystallography, University of Trieste, via L. Giorgieri 1, 34127 Trieste, Italy; nhickey@units.it (N.H.); sgeremia@units.it (S.G.)

**Keywords:** dihomooxacalix[4]arenes, naphthyl(thio)urea anion receptors, alkylammonium hydrochlorides, ditopic receptors, chiral recognition, NMR studies, UV-Vis absorption studies, fluorescence studies, X-ray diffraction, DFT calculations

## Abstract

Fluorescent dihomooxacalix[4]arene-based receptors **5a**–**5c**, bearing two naphthyl(thio)ureido groups at the lower rim via a butyl spacer, were synthesised and obtained in the cone conformation in solution. The X-ray crystal structures of 1,3- (**5a**) and 3,4-dinaphthylurea (**5b**) derivatives are reported. Their binding properties towards several anions of different geometries were assessed by ^1^H-NMR, UV-Vis absorption and fluorescence titrations. Structural and energetic insights of the naphthylurea **5a** and **5b** complexes were also obtained using quantum mechanical calculations. The data showed that all receptors follow the same trend, the association constants increase with the anion basicity, and the strongest complexes were obtained with F^−^, followed by the oxoanions AcO^−^ and BzO^−^. Proximal urea **5b** is a better anion receptor compared to distal urea **5a**, and both are more efficient than thiourea **5c**. Compounds **5a** and **5b** were also investigated as heteroditopic receptors for biologically relevant alkylammonium salts, such as the neurotransmitter γ-aminobutyric acid (GABA·HCl) and the betaine deoxycarnitine·HCl. Chiral recognition towards the guest *sec*-butylamine·HCl was also tested, and a 5:2 selectivity for (*R*)-*sec*-BuNH_3_^+^·Cl^−^ towards (*P*) or (*M*) enantiomers of the inherently chiral receptor 5a was shown. Based on DFT calculations, the complex [(*S*)-*sec*-BuNH_3_^+^·Cl^−^/(*M*)-5a] was indicated as the more stable.

## 1. Introduction

Calixarenes are among the most versatile macrocyclic compounds studied in supramolecular chemistry owing to their structural features [1,2]. They can be functionalized at the upper and lower rims, and they possess a pre-organized cavity available in different sizes and conformations. As a result, these compounds have been largely exploited as ion receptors.

Fluorescence spectroscopy, due to its high sensitivity, has been used for ion binding determination [3,4]. Fluorogenic moieties, such as naphthalene, anthracene and pyrene are among the most incorporated in the calixarene framework, leading to the development of fluorescent probes for anion and also ion-pair recognition. Examples of such fluorescent calix[4] arene [5,6,7,8,9,10], calix[5] arene [11] and calix[6]arene [12,13,14] receptors have been reported in the literature.

Anions play important roles in many biological and chemical systems, and also in the environment [15,16]. Synthetic anion receptors, namely calixarenes containing amide or (thio)urea groups interact exclusively through H-bonding with the anions. The NH groups provide strong and directional hydrogen bonds, resulting in well preorganized receptors. Some of these hosts can also act as ditopic receptors, simultaneously binding both ions of a given salt [17,18]. These receptors combine different binding sites in the same molecule, such as hydrogen bonds and oxygen donor atoms, besides an aromatic cavity that can establish π-CH interactions with the counter cation.

As part of our on-going interest on the host-guest properties of substituted dihomooxacalix[4]arenes (calix[4]arene analogues in which one CH_2_ bridge is replaced by one CH_2_OCH_2_ group) with (thio)urea units [19,20,21,22,23], we have extended our research into the study of fluorescent receptors for anion [24] and ion-pair recognition. Thus, dihomooxacalix[4]arene-based fluorescent sensors bearing (thio)urea groups as a binding site and naphthalene moieties as a fluorophore unit were obtained for the first time. This paper describes the synthesis of three disubstituted dihomooxacalix[4]arenes containing naphthylurea (compounds **5a** and **5b**) or naphthylthiourea (compound **5c**) residues at the 1,3- or 3,4-positions of the lower rim, via a butyl spacer. These derivatives were obtained in the cone conformation in solution, confirmed by NMR. The cone conformation was also observed in the solid state (for **5a** and **5b**) by X-ray diffraction. Their binding properties towards several relevant anions were assessed by proton NMR, UV-Vis absorption and fluorescence spectroscopy. The urea compounds (**5a** and **5b**) were also tested as heteroditopic receptors for biologically relevant alkylammonium salts, such as the amino acid γ-aminobutyric acid (GABA·HCl) and the betaine deoxycarnitine·HCl. GABA is an important neurotransmitter with inhibitory activity in mammal central nervous system. Deoxycarnitine results from enzymatic methylation of GABA, and as other betaines is used as an osmotic regulator in plants. Chiral recognition towards the chiral guest *sec*-butylamine·HCl was also investigated taking advantage of the intrinsic chirality of urea **5a**. Computational studies were also performed to add further insight to the binding process. 

## 2. Results and Discussion

### 2.1. Synthesis and Structural Analysis

**Scheme 1:**
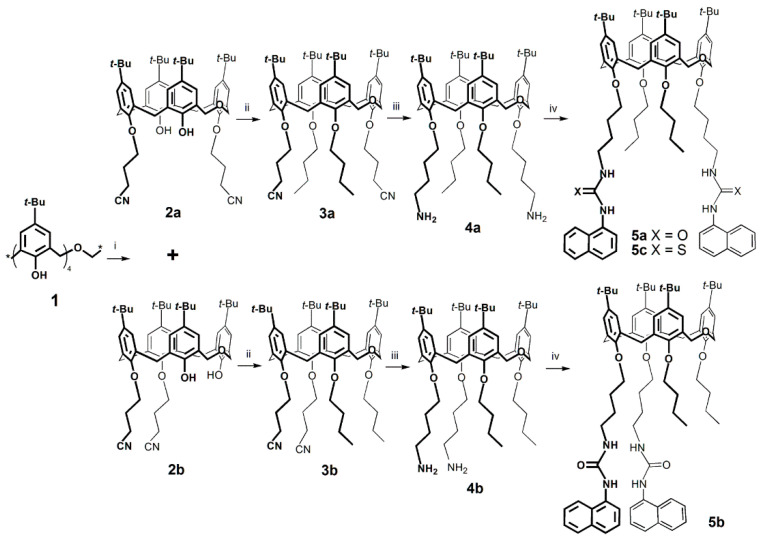
Synthesis of naphthyl(thio)ureas **5a**–**5c**. *Reaction conditions*: (i) Br(CH_2_)_3_CN, K_2_CO_3_, MeCN, Δ; (ii) *n*-BuI, NaH, THF/DMF, Δ; (iii) NaBH_4_/CoCl_2_, MeOH, rt; (iv) Naph-NCX, CHCl_3_, rt.

A few years ago we reported the reaction of parent compound **1** with bromobutyronitrile and K_2_CO_3_ to afford, after chromatographic separation, the asymmetric 1,3-dicyanodihydroxy derivative **2a** and the symmetric 3,4-dicyanodihydroxy derivative **2b** [19]. Following this synthetic route (Scheme 1), we undertook a three-step procedure from both the majority and the minority products **2a** and **2b**, respectively, obtaining in the last step naphthylurea **5a** and naphthylthiourea **5c** from the asymmetric diamine **4a** and naphthylurea **5b** from the symmetric **4b**. Comparing the ion affinity of receptors **5a** and **5b**, it is expected to obtain some insights about the role of the substitution pattern (distal vs. proximal) of the two ureido groups in a cooperative binding process.

The ^1^H-NMR spectra of inherently chiral receptors **5a** and **5c** in CDCl_3_ at room temperature show four singlets for the *tert*-butyl groups, five AB quartets for the CH_2_ bridge protons, four pairs of doublets for the aromatic protons of the calixarene skeleton, and two triplets and two singlets for the NHa and NHb protons, respectively. Beside these peaks, the spectra display also two triplets and several multiplets for the methyl and methylene protons of the *n*-butyl groups and butyl spacers, as well as for the aromatic protons of the naphthyl groups. The proton assignments were confirmed by COSY spectra. Receptors **5a** and **5c** were obtained in the cone conformation, as indicated by the three ArCH_2_Ar resonances in the range 29.5–30.8 ppm of the ^13^C-NMR spectra [25].

In contrast, receptor **5b** presents symmetric NMR spectra. The ^1^H-NMR spectrum displays two singlets for the *tert*-butyl groups, three AB quartets (in a 2:2:1 ratio) for the CH_2_ bridge protons, two pairs of doublets for the aromatic protons of the calixarene platform and one triplet and one singlet for the NHa and NHb protons, respectively, besides one triplet and several multiplets for the CH_3_ and CH_2_ protons of the butyl groups and spacers, and also for the aromatic protons of the naphthyl groups. The ^13^C-NMR spectrum exhibits two ArCH_2_Ar resonances at 30.3 ppm (two carbon atoms) and at 30.5 ppm (one carbon atom), indicating a cone conformation also for **5b**.

**Figure 1:**
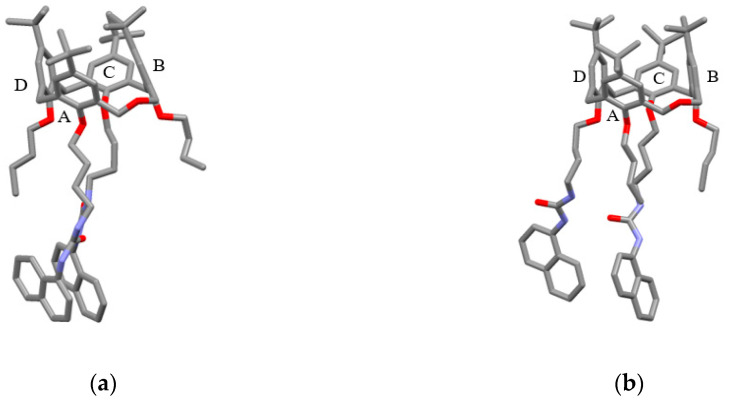
Solid state structures of (**a**) **5a** and (**b**) **5b**. The structures, with very similar cone conformations, show significant differences in conformation of the lower rim substituents. The atomic species are represented in CPK colours. Hydrogen atoms are omitted for clarity.

Small single crystals of naphthylureas **5a** and **5b** were analyzed using synchrotron radiation at 100 K. The X-ray structures confirm that both **5a** and **5b** adopt the expected cone conformation, also in the solid state. The structural model of **5a** clearly show that it is inherently chiral due to the 1,3-substitution pattern on the lower rim, which is asymmetric with respect to the dihomooxa bridge (Figure 1). As the space group is centrosymmetric, a racemic mixture of the two inherently chiral enantiomers is present in the crystals. With regard to the cone conformation, the planes of the two ureido-substituted phenyl rings **A** (connected to the dihomooxa bridge) and **C** make large dihedral angles of 124° and 143°, respectively, with respect to the mean plane of the methylene bridging groups. Angles greater than 90° indicate that the *tert*-butyl groups on the upper rims lean outwards from the centre of the cone (Figure 1). With respect to the butoxy-substituted phenyl rings, the plane of the one adjacent to the dihomooxa bridge (**B**) makes at a dihedral angle of 69° with the mean plane of the methylene bridging groups, with the upper rim inclined inwards. The last phenyl ring (**D**) is tilted slightly outwards, with a dihedral angle of 99°.

Consistently with what we have previously observed for analogous calixarenes containing ureido or thioureido units on the lower rim, the two ureido groups form a bifurcated intramolecular N–H···O hydrogen bond with N···O distances of 2.866 Å and 2.895 Å. In the present case, the hydrogen bond is quite symmetric, indicative of a strong interaction. The mean planes formed by the NCON atoms of the urea moieties show a dihedral angle of 24° (38° for the second orientation of the disordered naphthyl group of ring **A**), while the terminal naphthyl groups form dihedral angles of about 69° (Ring **A**, 86° for the second orientation) and 42° (Ring **C**) with respect to their corresponding planar NCON groups.

The overall result is that the two naphthyl groups are almost parallel, forming a dihedral angle of about 4° between their mean planes (7° for the second orientation) (Figure 1). The disordered naphthyl group (Ring **A**) is oriented head-to-tail (head-to-head in the second orientation) with respect to the other naphthyl group (Ring **C**). **5b** adopts a similar cone conformation (Figure 1). In contrast to **5a**, the 3,4-substitution pattern on the lower rim maintains the *C_s_* point symmetry of the macrocycle. Comparison of the dihedral angles of the four phenyl rings A, B, C and D with the mean plane of the methylene bridging groups, indicate an analogous cone conformation for **5a** and **5b** (Table 1). Thus, only slight differences are evident for the B (14°) and D (−11°) rings. For comparison, we have previously reported two analogous dihomooxacalix[4]arenes with a 1,3-substitution pattern on the lower rim and which differ from **5a** for the presence of two *p*-CF_3_-phenylurea moieties [23] or unsubstituted phenylurea moieties [20] in place of the naphthyl urea groups. All structures with the 1,3-substitution pattern on the lower rim exhibit conformations which are comparable (Table 1). Thus, the small difference in the cone conformation observed for **5b** can be attributed to the different substitution pattern. More significant difference between the two structures **5a** and **5b** are apparent in the relative orientations of the ureido substituents. The planes of the two naphthyl rings are almost perpendicular, with a dihedral angle of 88°, as opposed to the almost parallel situation found for **5a** (Table S1). There are significant differences in the hydrogen bonds formed by the various molecules discussed here (Table S2). **5a** and **5b** both form strong bifurcated intramolecular and intermolecular hydrogen bond, while for the *p*-CF_3_-Phurea moieties the intramolecular bonds are quite asymmetric, and in the case of the Phurea moieties a solvent molecule is involved and breaks the intermolecular H-bond pattern.

**Figure 2:**
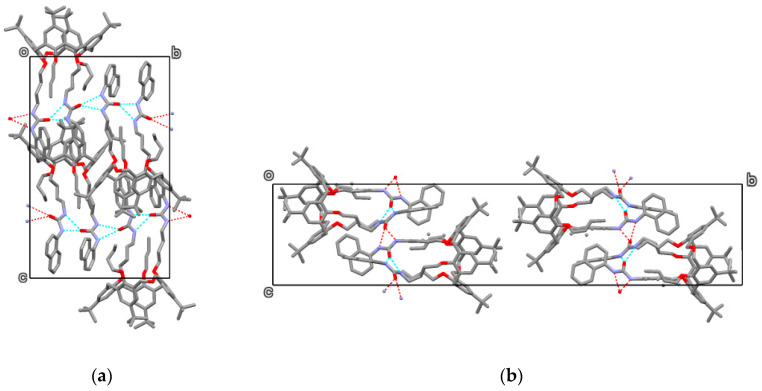
Crystal packing in the unit cells of (**a**) **5a** and (**b**) **5b**. In both cases the crystal packing is characterized by inversion-related antiparallel chains of bifurcated H-bonds. In **5a**, each molecule in the chain is generated by a 2_1_ screw operation and the chains are parallel to the *b*-axis. Each chain is composed of molecules with the same chirality and the antiparallel chain is composed of opposite chirality. In **5b**, the molecules in each chain are generated by the glide planes and the chains are parallel to the *a*-*c* cell diagonal. The atomic species are represented in CPK colours. Hydrogen atoms are omitted for clarity.

With regard to the crystal packing, in **5a** each molecule acts as both an *N*-donor (on one ureido group) and an *O*-acceptor (on the other ureido group) in the formation of two symmetry equivalent bifurcated intermolecular N–H···O hydrogen bonds (2.839 Å and 2.974 Å) with two other molecules generated by the 2_1_ screw axis symmetry operation, thereby forming alternating intramolecular /intermolecular H-bond chains parallel to the crystallographic *b*-axis. Two inversion-related anti- parallel chains are formed (Figure 2a). Each chain is composed of molecules with the same inherent chirality. Like **5a**, the urea group of **5b** forms an intramolecular bifurcated H-bond with N···O distances of 2.891 Å and 2.975 Å, and two intermolecular bifurcated H-bonds with N···O distances of 2.909 Å and 2.952 Å (Figure 2b). The intermolecular H-bonds are formed with molecules generated by the diagonal glide plane, forming antiparallel chains of H-bonds, parallel to the *a*-c cell diagonal.

### 2.2. Anion Complexation

#### 2.2.1. Proton NMR Studies

Complexation abilities of naphthyl(thio)ureas **5a**–**5c** toward relevant anions of different geometries (spherical, trigonal planar and tetrahedral) were investigated in CDCl_3_ by proton NMR titrations with tetrabutylammonium (TBA) salts. The association constants (as log *K*_ass_) were determined following the urea NH chemical shifts through the WinEQNMR2 program [26] and are reported in Table 2.

**Figure 3:**
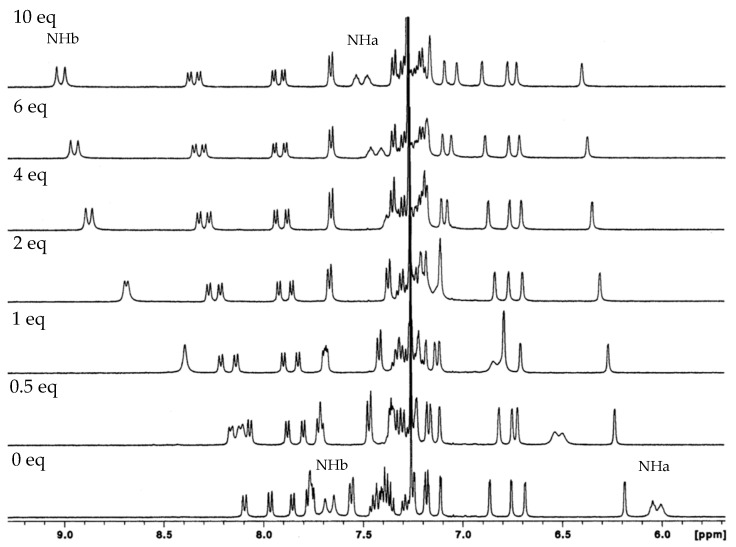
^1^H-NMR partial spectra (500 MHz, CDCl_3_, 25 °C) of Naph-urea **5a** with several equiv of TBA chloride.

Significant downfield shifts of the NH protons were observed upon addition of TBA salts to the receptors, clearly indicating hydrogen bonding interactions between the (thio)urea groups and the anions, as illustrated in Figure 3 and Figure S1. Only one set of signals was observed during the titrations, showing fast exchange rate between the free and the complexed receptor on the NMR time scale at room temperature. The titration curves obtained (Figure S2) evidence the 1:1 complexes, this stoichiometry being also confirmed by Job plots (Figures S3 and S4).

The comparison between naphthylureas **5a** and **5b** allowed us to make some considerations about the cooperative action of the two ureido moieties on alternate vs. adjacent positions of the calixarene framework. The results displayed in Table 1 indicate that proximal naphthylurea **5b** is a more efficient receptor for all the anions (except HSO_4_^−^). The association constants were, in average, 0.32 log units higher than those obtained for distal naphthylurea **5a** for the majority of the anions. In the case of H_2_PO_4_^−^ and AcO^−^ this enhancement was even higher (0.42 and 0.66 log units, respectively). A similar behaviour was previously observed with distal and proximal calix[4]arene diphenylurea analogues [27]. Concerning the spherical halides, the data reveal that both ureas **5a** and **5b** form the strongest complexes with F^−^ (log *K*_ass_ = 2.80 and 3.12, respectively), and the association constants increase with the anion basicity. With regard to the trigonal planar and tetrahedral anions, urea **5b** displays the same behaviour, showing the highest affinity for the carboxylate AcO^−^ and the inorganic oxoanion H_2_PO_4_^−^, respectively (log *K*_ass_ = 3.17 and 2.67). In the case of urea **5a**, and as observed before with other dihomooxa bidentate [19,23] and tetraurea receptors [21], there is a slight inversion of the basicity order (AcO^−^/BzO^−^ and H_2_PO_4_^−^/HSO_4_^−^). *π* stacking interactions may contribute to the increased binding of BzO^−^ over that of AcO^−^.

The anion binding results reported in Table 2 also show that naphthylthiourea **5c** is a weaker receptor than naphthylurea **5a**, despite the increased acidity of its NH groups. Thiourea **5c** exhibits however a similar trend to **5a**, with the anions bound according to their basicity. The association constants were, in average, 0.84 log units lower than those obtained for **5a**, except for the best bound anions F^−^, AcO^−^ and H_2_PO_4_^−^, whose differences were smaller (Δ log *K*_ass_ = 0.13, 0.34 and 0.23, respectively). Similar results were reported before for different homooxacalixarene thiourea receptors [21,28,29], as well as thioureido-calix[4] and [6]arene analogues [30,31]. This fact may be related to the larger size of sulfur atom, that destabilizes the cis-cis geometry required for anion binding, causing a lower preorganization and consequently a high energy demand of the thiourea groups compared to the urea ones [32].

The calixarene skeleton of symmetric urea **5b** seems to undergo no conformational changes upon the addition of 8 equiv of the salts, as the *tert*-butyl and the aromatic protons show very small downfield or upfield chemical shift variations (Δ*δ* ≤ 0.06 and 0.03 ppm, respectively). By contrast, asymmetric urea **5a** undergoes deeper conformational changes upon complexation. One of the four *t*-Bu groups experiences downfield variations from 0.05 to 0.20 ppm, while the other three display smaller upfield variations (from 0.01 to 0.14 ppm). The maximum chemical shifts were observed for BzO^−^ anion. Concerning the aromatic protons, some of them are overlapped by other peaks and difficult to follow during all the titration. However, it is possible to observe that two of them show significant downfield variations, from 0.10 to 0.29 ppm, the highest chemical shifts being observed for BzO^−^ (0.29 and 0.25 ppm).

#### 2.2.2. UV-Vis Absorption and Fluorescence Studies

**Figure 4:**
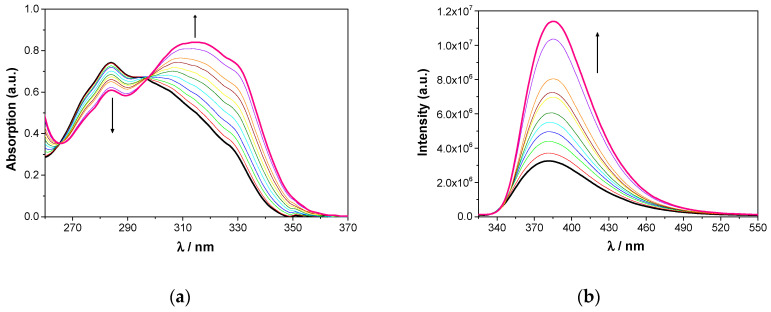
(**a**) Changes in the UV (**a**) and emission (**b**) spectra of Naph-urea **5a** (5.0 × 10^−5^ M) upon addition of TBA F (up to 10 equiv.) in CH_2_Cl_2_. The arrows indicate the decreasing or increasing amounts of salt.

The interactions between naphthyl(thio)ureas **5a**–**5c** and the previous anions as TBA salts have also been studied in dichloromethane by UV-Vis absorption and fluorescence titrations. Naphthylureas **5a** and **5b** showed identical behaviours with respect to anion complexation. Both ureas display absorption bands centred at approximately 283 nm in the absence of anions. These bands decrease in intensity upon addition of increasing amounts of F^−^, while a new one gradually appears at longer wavelength, reaching a maximum at approximately 315 nm (red shift of 32 nm). Isosbestic points can also be observed, as for example in Figure 4a for receptor **5a**. Concerning Cl^−^, AcO^−^, BzO^−^ and H_2_PO_4_^−^ anions similar absorption spectral changes were obtained (Figure S5), leading to red shifts of 20 nm, but with no isosbestic points. Finally, additions of Br^−^, NO_3_^−^ and HSO_4_^−^ anions induced progressive increases of the absorption, but no shifts in their maxima were recorded (Figure S6). Naphthylthiourea **5c** behaved differently for all the anions (Figure S7). In this case, the absorption band centred at 283 nm decreases as the anion concentration increases, presenting isosbestic points, but no significant shifts of its maximum.

With regard to steady-state fluorescence studies, receptors **5a** and **5b** exhibit emission bands centred at approximately 380 nm, characteristic of the naphthylurea groups [33]. Similar absorption and fluorescence spectra were reported in the literature for a ureido-calix[5]arene analogue [11]. No intramolecular excimer is observed in these fluorescence spectra, indicating the absence of *π*-π* stacking between the naphthyl moieties [33]. Both **5a** and **5b** display significant fluorescence lifetimes and quantum yields (Table 3). Successive additions of F^−^, AcO^−^ and H_2_PO_4_^−^ anions caused an increase of the emission intensity, as shown in Figure 4b. For Br^−^, NO_3_^−^ and HSO_4_^−^ anions this increase was less pronounced (Figure S8), and in the case of Cl^−^ and BzO^−^ (Figure S9) a quenching of the fluorescence intensity, with a concomitant decrease of the fluorescence quantum yield (Table 3), was observed. This decrease is stronger for asymmetric urea **5a**. In the case of Cl^−^ and **5a**, the fluorescence lifetime of the 1:1 complex shows a moderate decrease (about 2/3) with respect to the pristine receptor (Table 3), whereas the quantum yield drops by a factor of almost 4. This implies the existence of a marked static quenching in the complex, with only a few configurations being able to emit. The same applies to **5b**, although to a lesser extent. In the case of BzO^−^, the quenching is similar with respect to both intensity and lifetimes (Table 3). It is seen that the quenching arises mainly from an increase in the nonradiative decay constant in the complex, where aromatic moieties of receptor and anion appear to interact. The fluorescence of thiourea **5c** could not be studied in detail as this receptor is unstable upon irradiation.

Important spectral variations were observed for the three receptors in the presence of all the anions, allowing the calculation of the corresponding binding constants by absorption and emission (for the ureas) data (Table 4). The association constants are higher than those obtained by NMR (different concentration range), but follow the same trend. The more diluted solutions used in the UV/fluorescence titrations favour the dissociation of the salts, producing a higher concentration of the anions available for complexation and resulting in higher association constants [11]. Similar results were obtained by absorption and emission, in the same concentration range, showing that fluorescence can also be a useful method for the determination of the association constants. Proximal naphthylurea **5b** is a slightly better receptor for all the anions (except HSO_4_^−^) than distal urea **5a**, and F^−^, AcO^−^ and BzO^−^ are the best bound anions. Naphthylthiourea **5c** displayed the same trend as its urea analogue, with the anions bound according to their basicity (Table 4). However, **5c** is a weaker receptor, except in the cases of F^−^, AcO^−^ and H_2_PO_4_^−^ anions, for which it showed similar log *K*_ass_ values.

### 2.3. Organic Ion Pair Recognition

**Figure 5:**
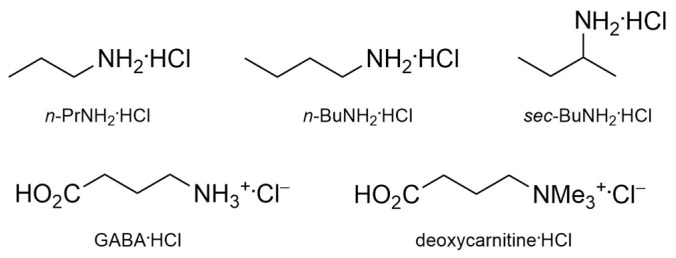
Structures of the ammonium hydrochloride guests studied.

Naphthylureas **5a** and **5b** have also been tested as ditopic receptors for *n*-propyl and *n*-butylammonium chlorides (Figure 5) in an exploratory study to estimate their ion pair binding efficiency.

**Figure 6:**
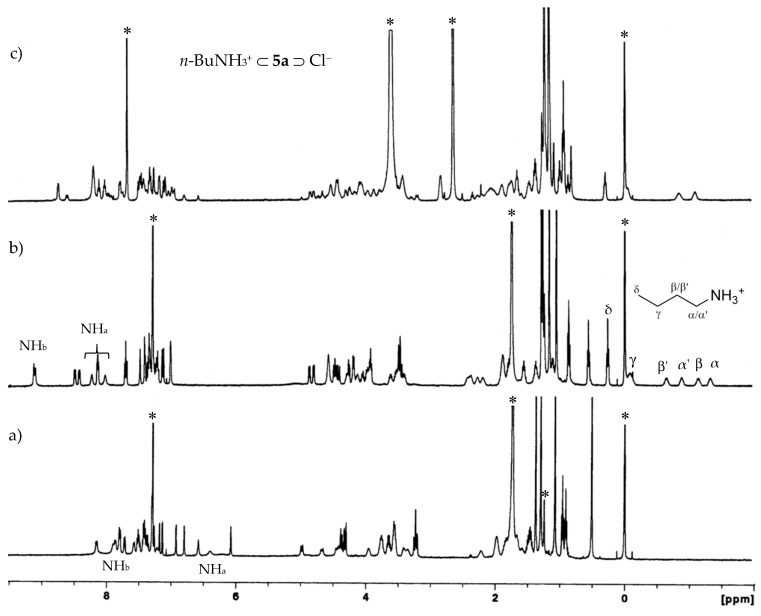
^1^H-NMR spectra (500 MHz) of: (**a**) [**5a**] = 1.0 mM at 253 K in CDCl_3_; (**b**) [**5a**] = [*n*-BuNH_2_·HCl] = 1 mM at 253 K in CDCl_3_; (**c**) [**5a**] = [*n*-BuNH_2_·HCl] = 1 mM at 243 K in CDCl_3_/DMSO-d_6_, 5:1, *v*/*v*. * Denotes residual solvent signals.

Proton NMR titrations were performed by adding increasing amounts (up to two equiv.) of the salts to CDCl_3_ solutions of **5a** and **5b** at room temperature. The addition of the first salt aliquot produced doubling of the receptor peaks, and also a new set of signals corresponding to the guest bound to the host. Alkylammonium cation inclusion inside the dihomoxa cavity is shown by the appearance of high field resonances for the alkyl groups (from −1.32 to −0.33 ppm for *n*-PrNH_3_^+^ and from −1.32 to 0.26 ppm for *n*-BuNH_3_^+^). In the case of receptor **5a**, due to its intrinsic chirality, the pairs of enantiotopic hydrogen atoms of the α- and β-CH_2_ groups of the included guest display chemically non-equivalent signals, as shown in Figure 6 for *n*-BuNH_3_^+^. On the other hand, chloride binding to the urea groups is demonstrated by the downfield shifts observed for all the NH protons (Δ*δ ≥* 1 ppm), indicating anion complexation through hydrogen-bond interactions. These observations are compatible with a slow binding process on the NMR time scale. The percentages of complex formation and the corresponding association constants could thus be determined by direct integration of the peaks. The temperature was lowered to 263 K/253 K to get a more sound integration of the signals, as they were slightly broad at room temperature. All host-guest pairs studied displayed percentages of complexation higher than 95% (corresponding to *K*_ass_ > 10^9^ M^−2^), preventing a more accurate calculation of the association constants in chloroform. 

These titration experiments were repeated in a more competitive solvent (CDCl_3_/DMSO-d_6_, 5:1) for the anion binding site and also that increases the ammonium solvation. It was necessary to lower the temperature until 273 K to observe the appearance of the peaks at the negative region of the ^1^H NMR spectra, and to lower until 243 K to a better integration of the signals. Thus, the results obtained (*K*_ass_ = 18,000 and 29,000 M^−2^, corresponding to 79% and 83% of complex formation for *n*-PrNH_3_^+^·Cl^−^ with **5a** and **5b**, respectively, and *K*_ass_ = 10,000 and 18,000 M^−2^, corresponding to 73% and 79% of complex formation for *n*-BuNH_3_^+^·Cl^−^ with **5a** and **5b**, respectively) show that receptor **5b** is more efficient than **5a** for both ion pairs, and the former guest is better bound than the latter by both receptors. This trend was also observed by the theoretical calculations (see below, Section 2.4). It is worth noting that in this solvent mixture the guests are less fixed within the aromatic cavity of the host, as indicated by the chemically equivalent signals for the α- and β-CH_2_ groups of the included guests (Figure 6c).

**Figure 7:**
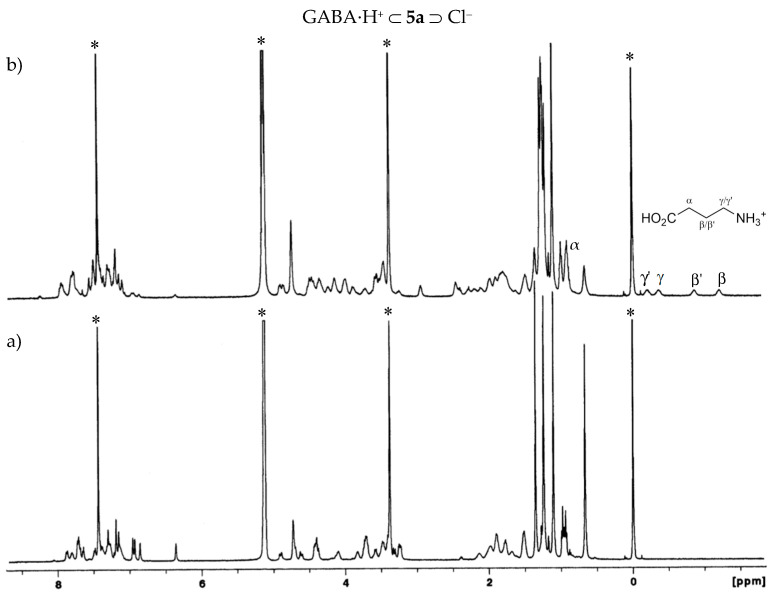
^1^H-NMR spectra (500 MHz, 233 K, CDCl_3_/CD_3_OD, 5:1, *v*/*v*) of: (**a**) [**5a**] = 1.0 mM; (**b**) [**5a**] = [GABA·HCl] = 1 mM. * Denotes residual solvent signals.

The binding affinities of ureas **5a** and **5b** were also extended to the aminoacid GABA·HCl and to the betaine deoxycarnitine·HCl (Figure 5) in a CDCl_3_/CD_3_OD (5:1, *v/v*) solvent mixture. The former guest was first tested in its zwitterionic form at room temperature and at 233 K. The NMR spectra remained almost unchanged after the addition of two equiv. of the guest, indicating no host-guest interaction. However, resonances at the negative region of the spectrum appeared when GABA·HCl was used, revealing the ammonium cation inclusion inside the aromatic cavity of the host (Figure 7).

For the inherently chiral receptor **5a**, four high field signals for the β- and γ-CH_2_ protons of the guest were observed (Figure S10), in analogy with the alkylammonium chloride cases seen previously. To obtain a more reliable integration of the signals, the NMR spectra were registered at 233 K. The data (81 and 88% of complex formation, corresponding to *K*_ass_ = 22,000 and 60,000 M^−2^ for **5a** and **5b**, respectively) indicate that receptor **5b** is more efficient than **5a**, being in line with the previous anion and alkylammonium chloride binding results. Concerning the latter guest, no interaction at all was detected with both receptors, suggesting that the more bulky groups (CH_3_ vs. H) of the betaine guest prevent the inclusion inside the macrocycle cavity.

**Figure 8:**
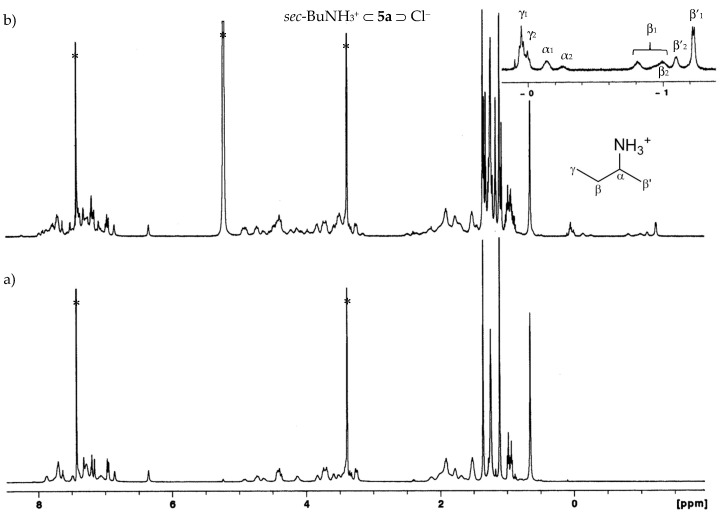
^1^H-NMR spectra (500 MHz, 223 K, CDCl_3_/CD_3_OD, 5:1, *v*/*v*) of: (**a**) [**5a**] = 1.0 mM; (**b**) [**5a**] = [*sec*-BuNH_2_·HCl] = 1 mM. Inset: 1 and 2 mean the two sets of signals for the *s*-Bu group of the guest inside the cavity for the two diastereotopic complexes formed. * Denotes residual solvent signals.

Chiral recognition towards racemic *sec*-butylamine·HCl guest (Figure 5) was also investigated with both urea receptors. The NMR binding studies (CDCl_3_/CD_3_OD, 5:1, *v*/*v*) performed at room temperature showed only a slight broadening of the host peaks. However, by lowering the temperature (until 223 K) it was possible to see the appearance of upfield resonances belonging to the guest inside the aromatic cavity of the hosts. In the case of the achiral urea **5b**, the addition of one equiv. of the guest gives rise to the appearance of an asymmetric structure. For example, six singlets for the *t*-Bu groups, corresponding to the free (two peaks) and to the complexed receptor (four peaks) can be observed. Five high field resonances for the *sec*-BuNH_3_^+^ guest, including two multiplets for the diastereotopic β methylene protons are also shown in the proton spectrum (Figure S11). The splitting of the *t*-Bu signals is not complete, preventing a very precise integration. However, a complex formation of approximately 70% could be determined. As reported before for the binding of *sec*-butylammonium ion with another achiral dihomooxacalixarene [34], the inclusion of the branched *sec*-BuNH_3_^+^ ion into the dihomooxa cavity should restrict its free motion, producing this chiral complex. Concerning racemic urea **5a** and in the same conditions as before, the proton NMR spectrum displays at least ten singlets for the *t*-Bu groups, four corresponding to the free host and the remaining ones corresponding to the two diastereotopic complexes formed [host(*P*)/guest(*R*) ≡ host(*M*)/guest(*S*) + host(*P*)/guest(*S*) ≡ host(*M*)/guest(*R*)] [35] (Figure 8). The percentage of complex formation is approximately of 65%. The same situation [host(*P*)/guest(*R*) + host(*M*)/guest(*R*)] was obtained when we used an enantiomerically pure guest [(*R*)-(−)-*sec*-butylamine·HCl]. Two sets of shielded resonances (8 signals instead of the expected 10 due to overlapping) for the *sec*-Bu group of the guest included into the cavity for the two diastereotopic complexes were seen in the high field region of the spectrum. A complete assignment of these peaks was obtained by a COSY spectrum (Figure S12). Their integration indicated a diastereomeric ratio of about 5:2.

By NMR is not possible to determine which complex [host(P)/guest(R) or host(M)/guest(R)] is more stable. Thus, DFT calculations were performed (see below) and showed an higher energy for the [host(M)/guest(*S*)] complex. On this basis, the more intense NMR signals were assigned to the more stable complex [(*S*)-*sec*-BuNH_3_^+^·Cl^−^/(*M*)-**5a**], that can be directly transferred to its enantiomeric pair [host(*P*)/guest(*R*)], and a selectivity of about 5:2 could be deduced.

### 2.4. Theoretical Studies

In order to get further insights into the anion binding ability of **5a** and **5b**, we performed quantum mechanical calculations on the complexed receptors with an extensive range of anions, including spherical halides (F^−^ vs. Cl^−^), trigonal (AcO^−^ vs. BzO^−^) and tetrahedral (HSO_4_^−^ vs. H_2_PO_4_^−^). Heteroditopic complexation properties of **5a** and **5b** were also studied with alkylammonium salts, comparing *n*-PrNH_3_^+^·Cl^−^ and *n*-BuNH_3_^+^·Cl^−^, and the affinity of the asymmetric host **5a** for the chiral guest *sec*-BuNH_3_^+^·Cl^−^ was also investigated.

**Figure 9:**
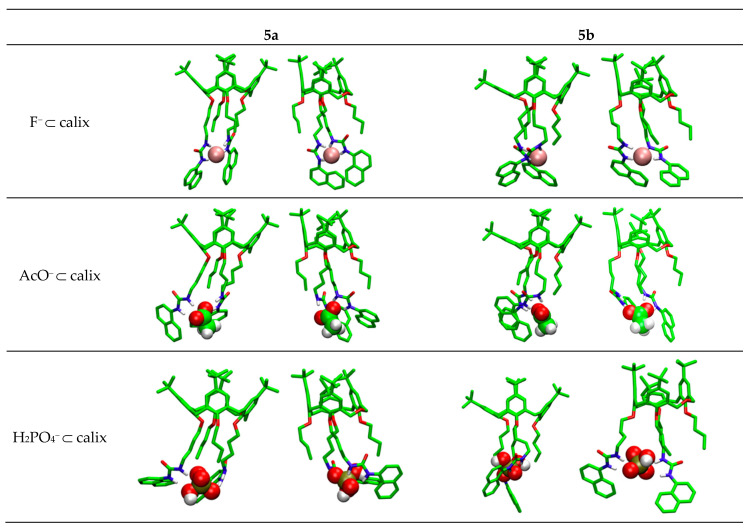
Structures of the X^−^ ⊂ Naph-urea complexes after QM optimization (orthogonal views). The atomic species are represented in CPK colours, except carbons that are in green.

Each anion, whatever is its geometry, is bonded to the urea groups of **5a** and **5b** via four hydrogen bonds, as illustrated by snapshots of the optimized structures for fluoride, acetate and hydrogenophosphate anions in Figure 9. Similar structures were found for the other anions, as shown in Figure S13. The urea moieties always interact with the coordinated anions that sit in a hole formed by the four hydrogens of the NH groups. AcO^−^ and BzO^−^ are recognized via their carboxylate groups, while the methyl and benzyl groups point away from the binding cavity. The tetrahedral anions interact via their non protonated oxygens.

The ∆*E* calculated complexation energies (∆*E* = *E*(complex) − *E*(free ligand) − *E*(ions), Table 5 and Table S3) nicely follow the association constants from Table 2. Naph-urea **5b** is always a better receptor than Naph-urea **5a**, but the ∆*E* differences depend on the nature of the anions and go from less than 15 kJ.mol^−1^ for HSO_4_^−^ to almost 60 kJ.mol^−1^ for the F^−^ ion. For the latter anion and for the trigonal planar the energy discrimination between **5a** and **5b** is quite high (more than 50 kJ.mol^−1^ for F^−^ and BzO^−^ and 30 kJ.mol^−1^ for AcO^−^), while it is smaller (less than 17 kJ.mol^−1^) for Cl^−^ and the tetrahedral anions. To analyse these differences the H-bond distances between the receptor and the anions were measured (Table S4). As expected, comparing the anions within the same geometry group, the averaged H-bond distances are correlated to the interaction energies: for the spherical F^−^ vs. Cl^−^ anions the mean values are 1.747 Å vs. 2.300 Å and 1.746 Å vs. 2.338 Å for **5a** and **5b**, respectively. This trend is also observed for the trigonal planar and tetrahedral ions. What is more surprising is the fact that the H-bond distances are equal or shorter for **5a**, although **5a** is always a weaker receptor than **5b**, for the same anion. Regarding the deformation energies of the calixarenes (i.e., the energy loss of the ligand) upon complexation (Table S5), it can be seen that the deformation is higher for **5a** than for **5b** with the spherical and trigonal planar anions (∆_def_*E* = 49 and 18 kJ.mol^−1^ for F^−^ and Cl^−^, respectively, and around 40 kJ.mol^−1^ for AcO^−^ and BzO^−^). These differences in the destabilisation of **5a** may explain its lower complexation energies for these anions, although its H-bond network is stronger. This tendency is inverted for the tetrahedral anions, the deformation being higher for **5b** than for **5a**, although this difference is smaller (Δ_def_*E* ≤ 13 kJ.mol^−1^). In this case, **5b** is much more destabilized (Table S5) than for the other groups of anions, presumably because the tetrahedral geometry of the anions is less adapted to the complexation by the two urea moieties.

**Figure 10:**
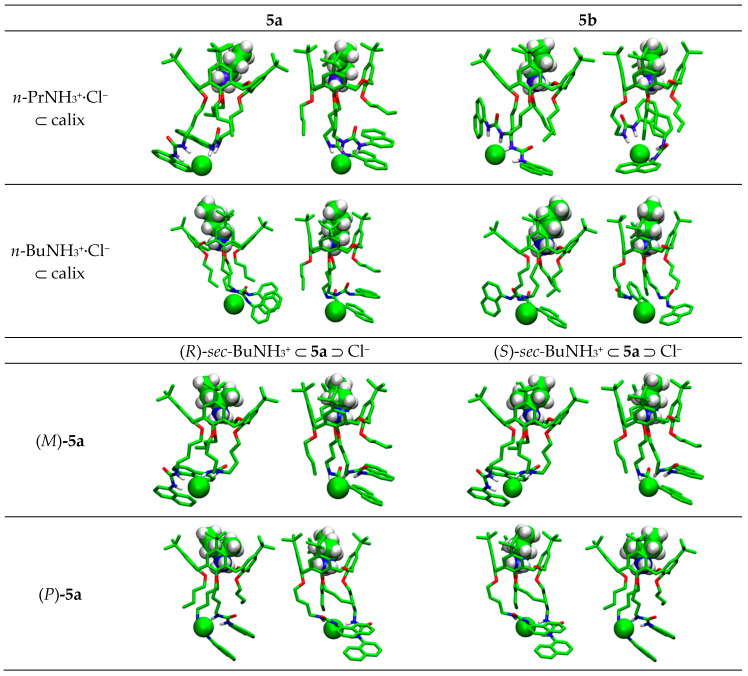
Structures of the R-NH_3_^+^ ⊂ **calix** ⊃ Cl^−^ complexes after QM optimization (orthogonal views). The atomic species are represented in CPK colours, except carbons that are in green.

Concerning the heteroditopic complexation with the alkylammonium salts, the position of the chloride anion is the same than for the single anion complexes (see Figure 10). The H-bonding network is similar: Cl^−^ interacts with the four hydrogen atoms of the urea groups with bond distances of about 2.3 Å each. The alkylammonium cations are positioned in the centre of the upper rim of the calixarenes. The ammonium group is asymmetrically H-bonded to the phenoxy oxygen atoms, as illustrated by the H-bond length given in Table S4. As NH_3_^+^ does not perfectly suit with the topology of the macrocycle cavity, it always displays two short H-bonds (less than 1.9 Å) and a longer one (up to 2.8 Å). The bridging ether oxygen atom is never involved in these interactions. The interaction energies obtained also indicate that **5b** is a better host than **5a**, and the *n*-PrNH_3_^+^·Cl^−^ salt is better bound than the *n*-BuNH_3_^+^·Cl^−^ one. Calculations with both enantiomers (*R*) and (*S*) of the chiral guest *sec*-BuNH_3_^+^·Cl^−^ show no clear differences in the coordination mode of receptor **5a**, displaying however higher energy for the latter enantiomer (Δ*E* = 8.3 and 4.3 kJ.mol^−1^ for (*M*) and (*P*) enantiomers of **5a**, respectively). The (*M*)-**5a** enantiomer leads to higher coordination energy than the (*P*)-**5a** one (∆*E* = 17 and 13 kJ·mol^−1^ for (*S*) and (*R*) guests, respectively), indicating the [(*S*)-*sec*-BuNH_3_^+^·Cl^−^/(*M*)-**5a**] complex as the most stable.

## 3. Materials and Methods

### 3.1. Synthesis

#### 3.1.1. Procedure for the Synthesis of (thio)ureas **5a** and **5c**

To a solution of **4a** [19] (0.77 g, 0.83 mmol) in CHCl_3_ (30 mL) was added 1.65 mmol of naphthyl isocyanate or naphthyl isothiocyanate, respectively. The mixture was stirred at room temperature under N_2_ for 4 h. Evaporation of the solvent yielded the crude products which were purified as described below.

*7,13,19,25-Tetra-tert-butyl-27,29-bis[[(N’-1-naphthylureido)butyl]oxy]-28,30-dibutoxy-2,3-dihomo-3-oxa- calix*[4]*arene* (**5a**). Flash chromatography (SiO_2_, eluent CH_2_Cl_2_/MeOH, from 99.5:0.5 to 95:5) followed by recrystallization from CH_2_Cl_2_/*n*-hexane: it was obtained in 30% yield (0.31 g); m.p. 258–259 °C; IR (KBr) 3314 cm^−1^ (NH), 1638 cm^−1^ (CO); ^1^H-NMR (CDCl_3_, 500 MHz) *δ* 0.58, 1.04, 1.28,1.36 [4s, 36H, C(CH_3_)_3_], 0.89, 0.94 (2t, 6H, *J* = 7.45 Hz, CH_3_), 1.45 (m, 4H, OCH_2_CH_2_C***H***_2_CH_3_), 1.64, 1.71, 1.82, 1.95, 2.13 (5m, 12H, OCH_2_C***H***_2_C***H***_2_CH_2_NH_a_ and OCH_2_C***H***_2_CH_2_CH_3_), 3.18, 4.33 (ABq, 2H, *J* = 13.9 Hz, ArCH_2_Ar), 3.20, 4.38 (ABq, 2H, *J* = 12.7 Hz, ArCH_2_Ar), 3.21, 4.35 (ABq, 2H, *J* = 12.9 Hz, ArCH_2_Ar), 3.35–3.62, 3.68, 3.76, 3.94 (several m, 12H, OC*H*_2_CH_2_CH_2_C***H***_2_NH_a_ and OC***H***_2_CH_2_CH_2_CH_3_), 4.45, 4.54 (ABq, 2H, *J* = 13.3 Hz, CH_2_OCH_2_), 4.48, 4.87 (ABq, 2H, *J* = 12.8 Hz, CH_2_OCH_2_), 6.01, 6.05 (2t, 2H, NH_a_), 6.19, 6.69, 6.76, 6.86, 7.11, 7.18, 7.19, 7.24 (8d, 8H, ArH), 7.29 (t, 1H, Napht), 7.35–7.47 (m, 5H, Napht), 7.56, 7.85, 7.97, 8.09 (4d, 5H, Napht), 7.65, 7.69 (2s, 2H, NH_b_), 7.76 (m, 3H, Napht); ^13^C-NMR (CDCl_3_, 125.8 MHz) δ 13.9, 14.1 (OCH_2_CH_2_CH_2_***C***H_3_), 19.35, 19.41 (OCH_2_CH_2_***C***H_2_CH_3_), 26.4, 26.7, 27.0, 28.5 (OCH_2_***C***H_2_***C***H_2_CH_2_NH_a_), 29.5, 30.7, 30.8 (ArCH_2_Ar), 31.2, 31.3, 31.6, 31.7 [C(***C***H_3_)_3_], 32.3, 32.6 (OCH_2_***C***H_2_CH_2_CH_3_), 33.7, 33.9, 34.15, 34.22 [***C***(CH_3_)_3_], 40.3, 40.6 (OCH_2_CH_2_CH_2_***C***H_2_NH_a_), 69.2 (2C) (CH_2_OCH_2_), 73.1, 74.0, 74.6, 75.4 (O***C***H_2_CH_2_CH_2_CH_2_NH_a_ and O***C***H_2_CH_2_CH_2_CH_3_), 119.7, 121.3, 121.5, 121.6, 123.6, 124.2 (2C), 124.4, 125.0, 125.2, 125.5, 125.7 (2C), 125.84, 125.87, 125.94, 126.0, 126.3, 126.8, 127.2, 128.3, 128.48 (ArH), 127.6, 128.52, 129.6, 131.8, 132.3, 132.6 (2C), 133.8, 134.1, 134.2 (3C), 134.3, 135.7, 144.4, 145.0, 145.1, 145.2, 152.4, 152.5, 153.0, 153.9 (Ar), 157.1, 157.6 (CO). Anal. Calcd for C_83_H_106_N_4_O_7_: C, 78.39; H, 8.40; N, 4.41. Found: C, 77.97; H, 8.68; N, 4.28.

7,13,19,25-Tetra-tert-butyl-27,29-bis[[(N’-1-naphthylthioureido)butyl]oxy]-28,30-dibutoxy-2,3-dihomo-3-

*oxa calix*[4]*arene* (**5c**). Flash chromatography (SiO_2_, eluent CH_2_Cl_2_/MeOH, from 99.5:0.5 to 97:3); the product obtained was chromatographed again (SiO_2_, eluent CH_2_Cl_2_/MeOH, from 99.7:0.3 to 97:3), yielding **5c** in 29% (0.31 g); m.p. 111–113 °C; ^1^H-NMR (CDCl_3_, 500 MHz) δ 0.75, 1.08, 1.11, 1.25 [4s, 36H, C(CH_3_)_3_], 0.87 (t, 6H, *J* = 7.45 Hz, CH_3_), 1.23–1.42, 1.54–1.87 (several m, 16H, OCH_2_C***H***_2_C***H***_2_CH_3_ and OCH_2_C***H***_2_C***H***_2_CH_2_NH_a_), 3.08, 4.26 (ABq, 2H, ArCH_2_Ar), 3.08, 4.28 (ABq, 2H, ArCH_2_Ar), 3.17, 4.24 (ABq, 2H, ArCH_2_Ar), 3.43–3.82 (several m, 12H, OC*H*_2_CH_2_CH_2_CH_3_ and OC*H*_2_CH_2_CH_2_C*H*_2_NH_a_), 4.30, 4.43 (ABq, 2H, CH_2_OCH_2_), 4.50, 4.53 (ABq, 2H, CH_2_OCH_2_), 6.02, 6,07 (2t br, 2H, NH_a_), 6.41, 6.87, 6.89, 6.99, 7.06, 7.07 (6d, 8H, ArH), 7.39–7.53 (several m, 8H, Napht), 7.57, 7.62 (2s, 2H, NH_b_), 7.82–7.96 (several m, 6H, Napht); ^13^C-NMR (CDCl_3_, 125.8 MHz) *δ* 14.10, 14.12 (OCH_2_CH_2_CH_2_***C***H_3_), 19.26, 19.34 (OCH_2_CH_2_***C***H_2_CH_3_), 25.7, 25.8, 27.3, 27.8 (OCH_2_***C***H_2_***C***H_2_CH_2_NH_a_), 29.7, 30.1, 30.7 (ArCH_2_Ar), 31.3, 31.4, 31.51, 31.54 [C(***C***H_3_)_3_], 32.2, 32.4 (OCH_2_***C***H_2_CH_2_CH_3_), 33.8, 33.9, 34.0, 34.1 [***C***(CH_3_)_3_], 45.7 (2C) (OCH_2_CH_2_CH_2_***C***H_2_NH_a_), 68.4, 68.5 (CH_2_OCH_2_), 73.4, 73.8, 74.1, 74.9 (O***C***H_2_CH_2_CH_2_CH_2_NH_a_ and O***C***H_2_CH_2_CH_2_CH_3_), 122.5 (2C), 123.4, 123.6, 124.9, 125.2, 125.3, 125.4, 125.5, 125.7, 125.8, 125.9, 126.2, 126.3, 127.0, 127.1, 127.4, 127.5, 128.5 (2C), 128.9, 129.0 (ArH), 129.96, 130.02, 130.5, 131.5, 131.8, 132.8, 132.9, 133.4, 133.5, 133.7, 134.6, 134.7 (2C), 144.5, 144.9 (2C), 145.1, 152.0, 152.1, 152.5, 153.2 (Ar), 181.6 (CS). Anal. Calcd for C_83_H_106_N_4_O_5_S_2_: C, 74.46; H, 8.19; N, 4.30; S, 4.92. Found: C 75.18; H, 8.72; N, 4.05; S, 4.25.

#### 3.1.2. Procedure for the Synthesis of Symmetric Urea **5b.** Precursor **2b** has Already been Obtained and **3b** and **4b** were Synthesised as Described for **3a** and **4a**

*7,13,19,25-Tetra-tert-butyl-28,29-bis[(cyanopropyl)oxy]-27,30-dibutoxy-2,3-dihomo-3-oxacalix*[4]*arene* (**3b**): Flash chromatography (SiO_2_, eluent gradient from *n*-hexane/ethyl acetate 95:5 to 90:10), 61% yield; RMN ^1^H (CDCl_3_, 500 MHz) δ 0.93, 1.19 [2s, 36H, C(CH_3_)_3_], 1.00 (t, 6H, *J* = 7.4 Hz, CH_3_), 1.45 (m, 4H, OCH_2_CH_2_C***H***_2_CH_3_), 1.76 (m, 4H, OCH_2_C***H***_2_CH_2_CH_3_), 2.28 (m, 4H, OCH_2_C***H***_2_CH_2_CN), 2.65 (m, 4H, OCH_2_CH_2_C*H*_2_CN), 3.24, 4.31 (ABq, 4H, *J* = 13.5 Hz, ArCH_2_Ar), 3.28, 4.26 (ABq, 2H, *J* = 13.0 Hz, ArCH_2_Ar), 3.62, 3.75 (2m, 4H, OC***H***_2_CH_2_CH_2_CH_3_), 3.84, 3.91 (2m, 4H, OC*H*_2_CH_2_CH_2_CN), 4.49, 4.68 (ABq, 4H, *J* = 13.5 Hz, CH_2_OCH_2_), 6.66, 7.00, 7.07 (3d, 8H, ArH); RMN ^13^C (CDCl_3_, 125,8 MHz) *δ* 14.1 (OCH_2_CH_2_CH_2_***C***H_3_), 14.4 (OCH_2_*C*H_2_CH_2_CN), 19.3 (OCH_2_CH_2_***C***H_2_CH_3_), 26.1 (OCH_2_CH_2_*C*H_2_CN), 29.7, 30.3 (ArCH_2_Ar), 31.4 [C(***C***H_3_)_3_], 32.4 (OCH_2_***C***H_2_CH_2_CH_3_), 34.0, 34.1 [***C***(CH_3_)_3_], 67.1 (CH_2_OCH_2_), 72.1, 74.9 (O***C***H_2_CH_2_CH_2_CN and O***C***H_2_CH_2_CH_2_CH_3_), 119.9 (CN), 123.2, 125.6, 125.8, 126.0 (ArH), 130.9, 132.8, 133.3, 133.7, 145.3, 145.6, 151.9, 152.2 (Ar). 

*7,13,19,25-Tetra-tert-butyl-28,29-bis[(aminobutyl)oxy]-27,30-dibutoxy-2,3-dihomo-3-oxacalix*[4] *arene* (**4b**): 0.83 g (87% yield) of product pure enough to be immediately used in the next step; RMN ^1^H (CDCl_3_, 500 MHz) δ 0.94, 1.18 [2s, 36H, C(CH_3_)_3_], 1.01 (t, 6H, *J* = 7.38 Hz, CH_3_), 1.49–1.60 (m, 8H, OCH_2_CH_2_C***H***_2_CH_3_ and OCH_2_CH_2_C***H***_2_CH_2_NH_a_), 1.79 (m, 4H, OCH_2_C***H***_2_CH_2_CH_3_), 1.98 (m, 4H, OCH_2_C*H*_2_CH_2_CH_2_NH_a_), 2.78 (t, 4H, OCH_2_CH_2_CH_2_C*H*_2_NH_a_), 3.17, 4.40 (ABq, 4H, *J* = 13.5 Hz, ArCH_2_Ar), 3.22, 4.39 (ABq, 2H, *J* = 13.0 Hz, ArCH_2_Ar), 3.60, 3.69 (2m, 4H, OC***H***_2_CH_2_CH_2_CH_3_), 3.78, 3.84 (2m, 4H, OC*H*_2_CH_2_CH_2_CH_2_NH_a_), 4.60, 4.66 (ABq, 4H, *J* = 13.5 Hz, CH_2_OCH_2_), 6.69, 6.96, 6.97, 7.05 (4d, 8H, ArH).

*7,13,19,25-Tetra-tert-butyl-28,29-bis[[(N’-1-naphthylureido)butyl]oxy]-27,30-dibutoxy-2,3-dihomo -3-oxacalix*[4]*arene* (**5b**). To a solution of **4b** (0.83 g, 0.89 mmol) in CHCl_3_ (35 mL) was added 0.26 mL (1.77 mmol) of naphthyl isocyanate. The mixture was stirred at room temperature under N_2_ for 4 h. Evaporation of the solvent yielded the crude product which was purified by flash chromatography (SiO_2_, eluent CH_2_Cl_2_/MeOH, from 99.7:0.3 to 98:2), followed by recrystallization from CH_2_Cl_2_/*n*-hexane: it was obtained in 40% yield (0.45 g); mp 211–213 °C; IR (KBr) 3329 cm^−1^ (NH), 1647 cm^−1^ (CO); ^1^H-NMR (CDCl_3_, 500 MHz) δ 0.87 (t, 6H, *J* = 7.35 Hz, CH_3_), 0.95, 1.17 [2s, 36H, C(CH_3_)], 1.42 (m, 4H, OCH_2_CH_2_C*H*_2_CH_3_), 1.70 (m, 8H, OCH_2_C*H*_2_CH_2_CH_3_ and OCH_2_CH_2_C*H*_2_CH_2_NH_a_), 1.98, 2.06 (2m, 4H, OCH_2_C*H*_2_CH_2_CH_2_NH_a_), 3.17, 4.33 (ABq, 4H, *J* = 13.1 Hz, ArCH_2_Ar), 3.20, 4.39 (ABq, 2H, *J* = 12.9 Hz, ArCH_2_Ar), 3.34, 3.43 (2m, 4H, OCH_2_CH_2_CH_2_C*H*_2_NH_a_), 3.56, 3.72 (2m, 4H, OC*H*_2_CH_2_CH_2_CH_2_NH_a_), 3.74, 3.77 (2m, 4H, OC*H*_2_CH_2_CH_2_CH_3_**)**, 4.52, 4.61 (ABq, 4H, *J* = 13.4 Hz, CH_2_OCH_2_), 5.83 (t, 2H, NH_a_), 6.70, 6.95, 7.03 (3d, 8H, ArH), 7.37, 7.38, 7.43 (3t, 6H, Napht), 7.48 (s, 2H, NH_b_), 7.61, 7.74, 7.81, 8.01 (4d, 8H, Napht); ^13^C-NMR (CDCl_3_, 125.8 MHz) δ 14.1 (OCH_2_CH_2_CH_2_***C***H_3_), 19.3 (OCH_2_CH_2_*C*H_2_CH_3_), 27.3, 27.9 (OCH_2_***C***H_2_***C***H_2_CH_2_NH_a_), 30.3, 30.5 (ArCH_2_Ar), 31.5 [C(***C***H_3_)_3_], 32.6 (OCH_2_***C***H_2_CH_2_CH_3_), 34.0 [***C***(CH_3_)_3_], 40.7 (OCH_2_CH_2_CH_2_***C***H_2_NH_a_), 67.4 (CH_2_OCH_2_), 74.21, 74.24 (O***C***H_2_CH_2_CH_2_CH_2_NH_a_ and O***C***H_2_CH_2_CH_2_CH_3_), 121.1, 121.6, 123.2, 125.2, 125.56, 125.59, 125.61, 125.97, 126.04, 126.1, 128.5 (ArH), 128.2, 131.0, 133.2, 133.3, 133.7, 134.1, 134.4, 144.8, 145.0, 152.3, 153.0 (Ar), 157.3 (CO). Anal. Calcd for C_83_H_106_N_4_O_7_: C, 78.39; H, 8.40; N, 4.41. Found: C,78.51: H, 8.38; N, 4.40).

### 3.2. Determination of the Crystallographic Structures of **5a** and **5b**

Single crystals suitable for an X-ray investigation were obtained by slow evaporation of solutions containing compound **5a** and **5b** using dichloromethane/ethanol solvent mixtures. Data collection was carried out at the XRD1 beamline of the Elettra synchrotron (Trieste, Italy), employing the rotating-crystal method with a Dectris Pilatus 2M area detector (DECTRIS Ltd., Baden-Daettwil, Switzerland). Single crystals were dipped in paratone cryoprotectant, mounted on a loop and flash-frozen under a liquid nitrogen stream at a 100 K. Diffraction data were indexed and integrated using the XDS package [36], while scaling was carried out with XSCALE [37]. Structures was solved using the SHELXT program [38] and structure refinement was performed with SHELXL-14 [39], operating through the WinGX GUI [40], by full-matrix least-squares (FMLS) methods on F^2^. Non-hydrogen atoms with occupancy of more than 50% were anisotropically refined, while non-hydrogen atoms with a lower occupancy were refined isotropically. Hydrogen atoms were added at the calculated positions and refined using the riding model. Crystallographic data and refinement details are reported in Table S6.

### 3.3. H-NMR Titrations

The anion association constants (as log *K*_ass_) were determined in CDCl_3_ by ^1^H-NMR titration experiments. Several aliquots (up to 10 equiv.) of the anion solutions (as TBA salts) were added to 0.5 mL solution of the receptors (2.5 × 10^−3^ −5 × 10^−3^ M) directly in the NMR tube. The spectra were recorded after each addition of the salts, and the temperature of the NMR probe was kept constant at 25 °C. The association constants were evaluated using the WinEQNMR2 program [26] and following the urea NH chemical shifts. The Job methods were performed keeping the total concentration in the same range as before. In the case of ion-pair recognition studies, the percentage of complexation was determined by direct ^1^H-NMR integration of the free and complexed peaks of the hosts and/or the guests, present at equilibrium. The samples were prepared by mixing aliquots of stock solutions of the host (600 μL) and guests (60 μL) to obtain a final equimolar host-guest solution of 1.0 × 10^−3^ M. For each host-guest system titrations were repeated at least two times. Details related to these experiments have already been described [20].

### 3.4. UV-Vis Absorption and Fluorescence Studies

Absorption and fluorescence studies were done using an UV-3101PC UV-Vis-NIR spectrophotometer (Shimadzu, Kyoto, Japan) and a Fluorolog F112A fluorimeter (Spex Industries, Edison, NJ, USA) in right-angle configuration, respectively. The association constants were determined in CH_2_Cl_2_ by UV-Vis absorption spectrophotometry and by steady-state fluorescence at 25 °C. The absorption spectra were recorded between 260 and 370 nm and the emission ones between 325 and 550 nm, and using quartz cells with an optical path length of 1 cm. Several aliquots (up to 10 equiv) of the anion solutions (as TBA salts) were added to a 2 mL solution of the receptors (3.0 × 10^−5^–5.0 × 10^−5^ M) directly in the cell. The spectral changes were interpreted using the HypSpec 2014 program [41]. Details concerning the photophysical properties determination has already been described [23].

### 3.5. Quantum Chemistry Calculations

Stationary points were optimized with the Gaussian 09 program [42] with the B3LYP [43] density functional with the 6-31G(d,p) basis set. A D3-Grimme correction [44] was also used. Experimental X-ray diffraction structure determinations were employed as the starting structures for the calixarene hosts and different starting positions for the ions were used for the geometry optimization. All reported structures were confirmed as energy minima, with no negative eigenvalue in the Hessian matrix. The structures and energies given are the most stable conformations obtained after optimization. The interaction energy Δ*E* between the calixarenes and the ions (Δ*E* = *E*(complex) − *E*(free calix) − *E*(ion)) was calculated with respect to the optimized geometries of all species.

## 4. Conclusions

New fluorescent dihomooxacalix[4]arene receptors containing two (thio)urea moieties in distal and proximal positions (1,3-dinaphthylurea **5a**, 3,4-dinaphthylurea **5b** and 1,3-dinaphthylthiourea **5c**) at the lower rim linked by a butyl spacer were obtained in the cone conformation in solution. The X-ray structures of **5a** and **5b** were reported and revealed only small differences in the cone conformation. The main difference in the structures is ascribable to the mutual orientation of the naphthyl rings of the ureido substituents, which are almost parallel in **5a** and almost perpendicular in **5b**. Both crystal structures are characterized by intra- and inter-molecular bifurcated H-bonds involving the ureido groups. The anion binding affinity of these derivatives was established by ^1^H NMR, UV-Vis, fluorescence and DFT studies. 1:1 complexes between anions of different geometries and the receptors through hydrogen bonding were obtained. The results revealed that for all receptors the association constants increase with the anion basicity, and the strongest complexes were obtained with F^−^, followed by the carboxylates AcO^−^ and BzO^−^. Symmetric urea **5b** is a better anion receptor compared to the asymmetric urea **5a**, as shown by all the spectroscopic techniques used and corroborated by the quantum mechanical calculations. Both ureido compounds are more efficient than thiourea **5c**. As ditopic receptors, ureas **5a** and **5b** showed a very high affinity for the guests *n*-PrNH_3_^+^·Cl^−^ and *n*-BuNH_3_^+^·Cl^−^ (*K*_ass_ = 1.0 × 10^4^ − 2.9 × 10^4^ M^−2^ in CDCl_3_/DMSO-d_6_, 5:1), as well as for the neurotransmitter GABA·HCl (*K*_ass_ = 2.2 × 10^4^ and 6.0 × 10^4^ M^−2^ respectively, in CDCl_3_/CD_3_OD, 5:1). The bulkier CH_3_ groups of the betaine guest prevented its inclusion inside the macrocycle cavity. Concerning chiral recognition, the enantiopure (*R*)-*sec*-BuNH_3_^+^·Cl^−^ guest displayed a 5:2 selectivity towards (*P*) and (*M*) enantiomers of the inherently chiral host **5a**. Based on DFT calculations, the [(*S*)-*sec*-BuNH_3_^+^·Cl^−^/(*M*)-**5a**] complex was deduced as the more stable.

## Figures and Tables

**Table 1:** Comparison of cone conformations: Dihedral angles between corresponding aryl planes of the calixarene cones (A, B, C and D) and the mean planes of the bridging methylene carbon atoms for various dihomooxacalix[4]arenes.

	A (°)	B (°)	C (°)	D (°)
**5a**	124 ^a^	69	143 ^a^	99
**5b**	125	83	140 ^a^	88 ^a^
*p*-CF_3_-Phurea ^b^	125 ^a^	66	131 ^a^	101
Phurea ^c^ (I)	123 ^a^	64	133 ^a^	100
Phure a^c^ (II)	121 ^a^	74	137 ^a^	97

^a^ Ureido substituent on lower ring; ^b^ data taken from ref. 23; ^c^ data taken from ref. 20 for two independent molecules in the asymmetric unit. See Figure **2** and text for labelling of rings A, B, C and D.

**Table 2:** Association constants (log *K*_ass_) ^a^ of dihomooxa naphthyl(thio)ureas **5a**-**5c** in CDCl_3_ at 25 °C.

	Spherical				Trigonal Planar			Tetrahedral	
	F^−^	Cl^−^	Br^−^	I^−^	NO_3_^−^	AcO^−^	BzO^−^	HSO_4_^−^	H_2_PO_4_^−^
I. Radius/Å ^b^	1.33	1.81	1.96	2.20	1.79	2.32	—	1.90	2.00
**5a**	2.80	2.60	2.12	1.78	2.12	2.51	2.76	2.54	2.25
**5b**	3.12	2.91	2.46	1.94	2.38	3.17	3.07	2.52	2.67
**5c**	2.67	1.75	1.06	1.09	1.06	2.17	2.01	1.89	2.02

^a^ Estimated error < 10%; ^b^ Data quoted in Marcus, I. *Ion Properties*; Marcel Dekker: New York, pp. 50–51,1997.

**Table 3:** Photophysical properties of Naphureas **5a** and **5b** in CH_2_Cl_2_ at 25 °C.

	*λ*_max,abs_ (nm)	***ε*** **(M** **^−1^ cm^−1^)**	*λ*_max,em_ (nm)	Stokes Shift ^a^ (nm)	*τ*_f_ (ns)	*Φ*_F_	*k*_r_ (ns^−1^)	*k*_nr_ (ns^−1^)
**5a**	283	$1.5\times 10^{4}$	382	99	8.93	0.31 ^b^	0.035	0.077
**5a** + Cl^−^	303	$2.4\times 10^{4}$	382	79	6.26	0.085 ^b^	—	—
**5a** + BzO^−^	303	$2.0\times 10^{4}$	382	79	1.98	0.048 ^b^	0.024	0.48
**5b**	282	$1.8\times 10^{4}$	379	97	7.57	0.26 ^b^	0.034	0.098
**5b** + Cl^−^	302	$2.3\times 10^{4}$	379	77	6.63	0.16 ^b^	—	—
**5b** + BzO^−^	302	$1.7\times 10^{4}$	379	77	3.27	0.078 ^b^	0.024	0.28

^a^ Computed as $\lambda_{\max,\mathrm{em}}-\lambda_{\max,\mathrm{abs}}$; ^b^ Against quinine sulfate *Φ*_F_ = 0.546 in H_2_SO_4_ 0.5 M.

**Table 5:** B3LYP/6-31G(d.p) + BG3BJ complexation energies ∆*E* (in kJ.mol^−1^) for the guest-host complexes.

	∆*E* (kJ.mol^−1^)	
Host	**5a**	**5b**
F^−^	−498.9	−557.1
Cl^−^	−202.5	−219.0
AcO^−^	−261.5	−291.4
BzO^−^	−232.1	−283.3
HSO_4_^−^	−192.3	−206.2
H_2_PO_4_^−^	−220.6	−236.6
*n*-PrNH_3_^+^·Cl^−^	−736.3	−811.6
*n*-BuNH_3_^+^·Cl^−^	−656.5	−709.5
(*R*)-*sec-*BuNH_3_^+^·Cl^−^/(*M*)-**5a**	−704.1	—
(*S*)-*sec*-BuNH_3_^+^·Cl^−^/(*M*)-**5a**	−712.4	—
(*R*)-*sec-*BuNH_3_^+^·Cl^−^/(*P*)-**5a**	−691.1	—
(*S*)-*sec*-BuNH_3_^+^·Cl^−^/(*P*)-**5a**	−695.4	—

**Table 4:** Association constants (log *K*_ass_) ^a^ of dihomooxa naphthyl(thio)ureas **5a**–**5c** in C H_2_Cl_2_ at 25 °C.

		Spherical			Trigonal Planar			Tetrahedral	
		F^−^	Cl^−^	Br^−^	NO_3_^−^	AcO^−^	BzO^−^	HSO_4_^−^	H_2_PO_4_^−^
**5a**	Abs	4.21	3.59	3.20	3.19	3.79	3.94	3.13	3.06
	Emi	4.05	3.48	3.14	3.12	3.66	4.00	3.01	2.90
**5b**	Abs	4.36	3.69	3.37	3.31	4.16	4.08	2.90	3.16
	Emi	4.34	3.67	3.23	3.23	4.21	3.84	2.97	3.14
**5c**	Abs	4.18	3.01	2.71	3.07	3.66	3.71	2.87	3.14

^a^ Estimated error < 10%.

## Supplementary Materials

The following are available online: Crystallographic data and refinement details; titration curves with TBA salts in CDCl_3_; Job’s plots; absorption and emission spectra with TBA salts; COSY spectra of **5a** + GABA·HCl and **5a** + *sec*-BuNH_2_·HCl; ^1^H, ^13^C and COSY spectra of compounds **3b**, **5a**, **5b** and **5c**.
