# Supplementary material for: Dihomooxacalix[4]arene-Based Fluorescent Receptors for Anion and Organic Ion Pair Recognition
Source: Molecules. 2020 Oct 14;25(20):4708. doi: 10.3390/molecules25204708 (PMC7587342; doi:10.3390/molecules25204708)
Supplement: Supplementary file 1 [file molecules-25-04708-s001.pdf]

Supporting Information

Dihomooxacalix[4]arene-Based Fluorescent Receptors for Anions and Organic Ion Pairs Recognition

Alexandre S. Miranda,[1,2] Paula M. Marcos,[1,3,*] José R. Ascenso,[4] Mário N. Berberan-Santos,[2] Rachel Schurhammer,[5] Neal Hickey[6] and Silvano Geremia[6]

List of contents

1. Dihedral angles and H-bond interactions for various dihomooxacalix[4]arenes 2
2. Partial spectra of Naph-urea **5b** with TBA Cl 4
3. Titration curves of naphthyl ureas with TBA salts in $CDCl_3$ 5
4. Job's plot based on ^{1}H NMR data 6
5. Absorption spectra of naphthyl (thio)ureas with TBA salts in CH_2Cl_2 8
6. Emission spectra of naphthyl ureas with TBA salts in CH_2Cl_2 10
7. Section of the COSY spectrum of **5a** + GABA·HCl 11
8. ^{1}H NMR spectra of **5b** + *sec*-BuNH$_2$·HCl 12
9. Section of the COSY spectrum of **5a** + *sec*-BuNH$_2$·HCl 13
10. Quantum mechanical calculations data 13
11. Crystallographic data and refinement details 17
12. ^{1}H NMR spectra of **3b**, **5a**, **5b** and **5c** 18
13. ^{13}C NMR spectra of **3b**, **5a**, **5b** and **5c** 22
14. COSY spectra of **3b**, **5a**, **5b** and **5c** 26

Table S1. Comparison of a naphthylurea group conformations: Dihedral angles between the mean planes of the NCON atoms and the mean planes of the naphthyl carbon atoms for various dihomooxacalix[4]arenes

	NCON-NCON (°)	NCON-Naphthyl (°)	NCON-Naphthyl (°)	Naphthyl – Naphthyl (°)
**5a**	24	69 (A)	42 (C)	4
**5b**	69	40 (C)	41 (D)	88
***p*-CF_3-Phurea**[a]	28	20 (A)	40 (C)	74
**Phurea**[b]	11	45 (C)	48 (A)	3
	87	3 (C)	34 (A)	72

[a] Data taken from ref. 23; [b] Data taken from ref.20. In this case there were 2 independent molecules in the asymmetric unit. See Figure 2 and text for description of rings A, C and D.

Table S2. Comparison of hydrogen bonding interactions found in various dihomooxacalix[4]arenes

	D-H•••A[a]	d(D•••A) (Å)
		Intramolecular
**5a**	N(1C)-H•••O(2A)	2.866
	N(1C)-H•••O(2A)	2.895
**5b**	N(1C)-H•••O(2B)	2.891
	N(2C)-H•••O(2B)	2.975
***p*-CF₃-Phurea**[b]	N(2C)-H•••O(2A)	2.89
	N(1C)-H•••O(2A)	3.06
**Phurea**[c] **(I)**	N(1n)-H•••O(3p)	2.892
	N(1q)-H•••O(3p)	2.843
**(II)**	N(5na)-H•••O(7pa)	3.11
	N(5q)-H•••O(7pa)	2.80
		Intermolecular
**5a**	N(1A)-H•••O(2C)	2.839
	N(2A)-H•••O(2C)	2.974
**5b**	N(2B)-H•••O(2C)	2.909
	N(1B)-H•••O(2C)	2.952
***p*-CF₃-Phurea**[b]	N(1A)-H•••O(2C)	2.85
	N(2A)-H•••O(2C)	3.16
**Phurea**[c]	N(3n)-H•••O(5p)	2.872
	N(3q)-H•••O(5p)	2.905
	N(7na)-H•••O(1pa)	2.55

[a] Atom labels are indicated in parenthesis; [b] Data taken from ref. 23; [c] Data taken from ref.20. In this case there were 2 independent molecules in the asymmetric unit. See Figure 2 and text for description of rings A, C and D.

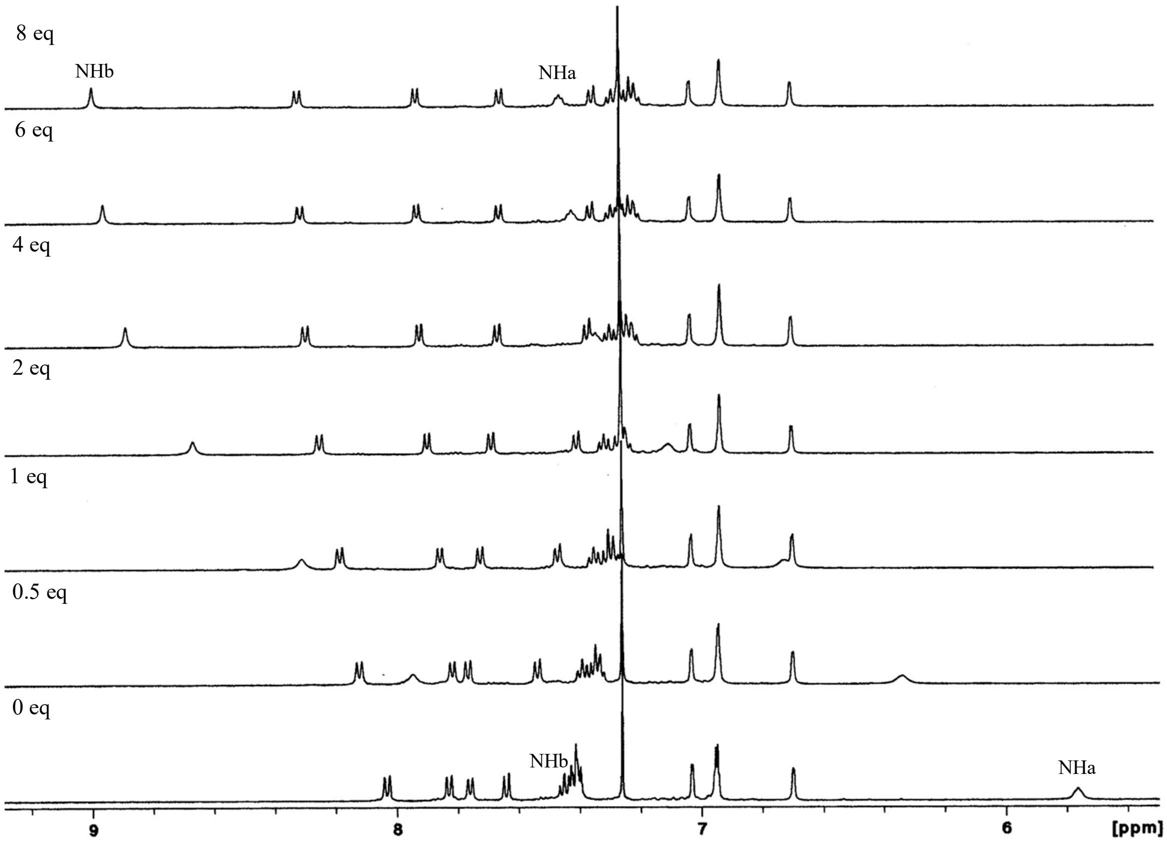

Figure S1. ^{1}H NMR partial spectra (500 MHz, $CDCl_3$, 25 ºC) of Naph-urea **5b** with several equiv of TBA chloride.

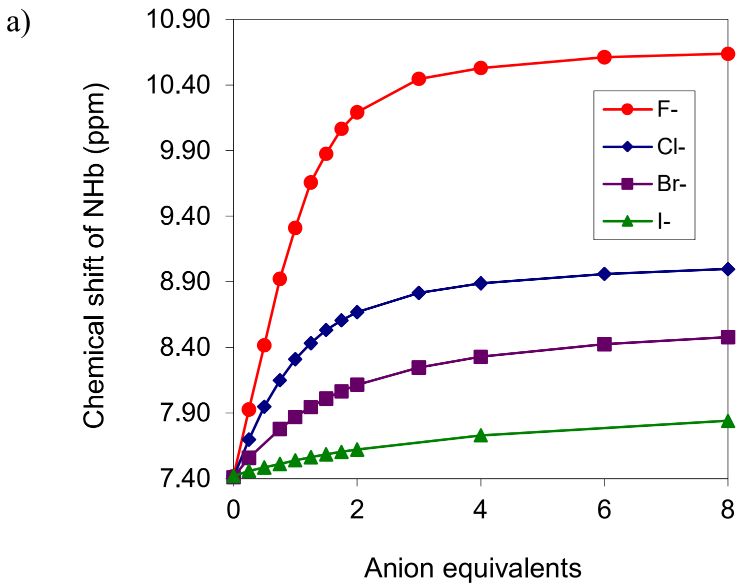

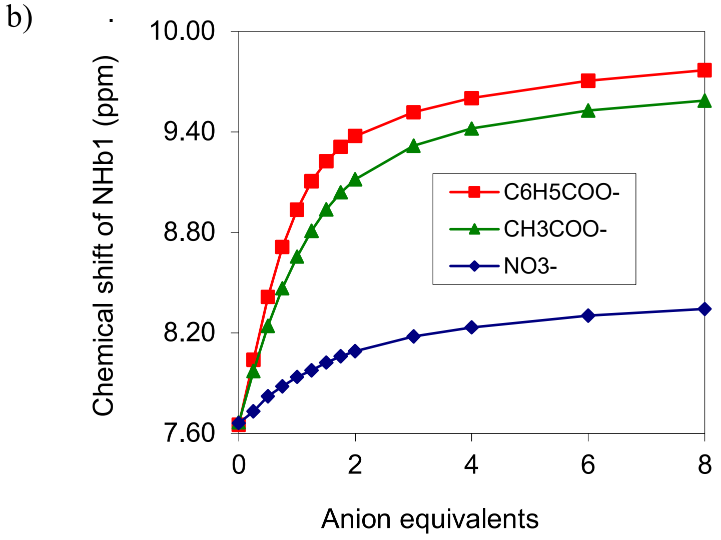

Figure S2. Titration curves of (a) Naphurea **5b** and (b) Naphurea **5a** with TBA salts in $CDCl_3$.

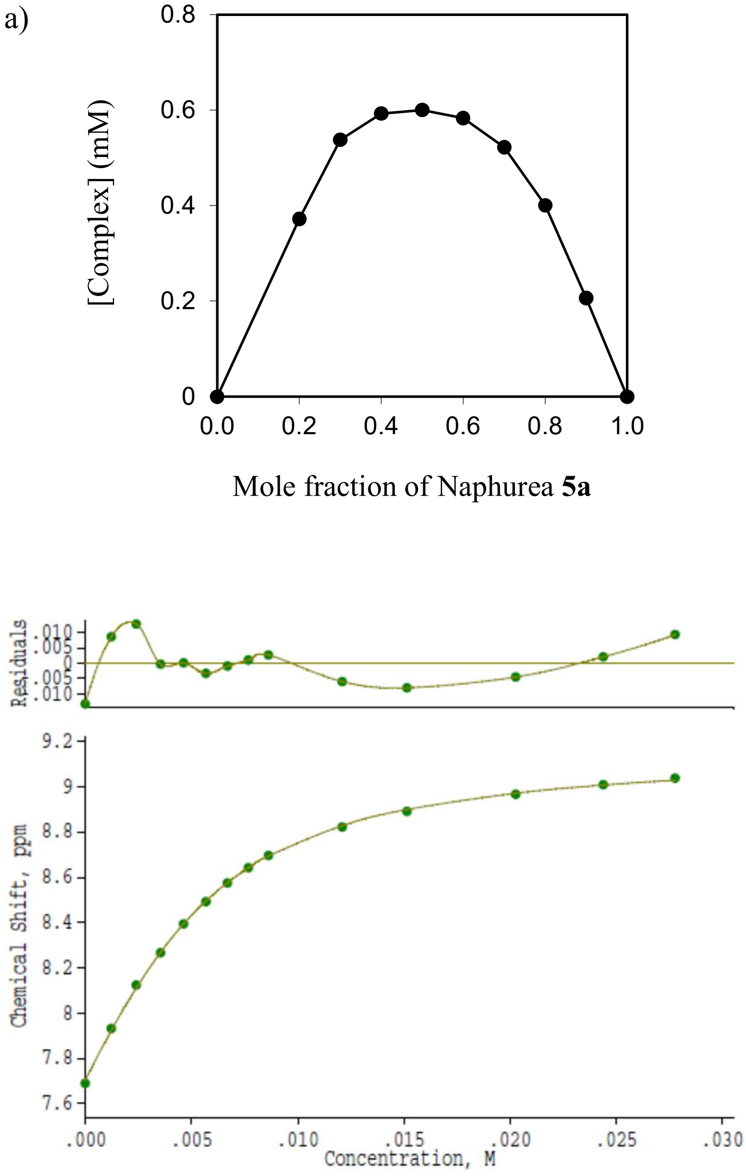

Figure S3. (a) Job plot based on ^{1}H NMR data for Naphurea **5a** + Cl^-, (b) Titration data fitted with 1:1 model and residual distribution. Total concentration 2.5×10^{-3} M in $CDCl_3$.

a)

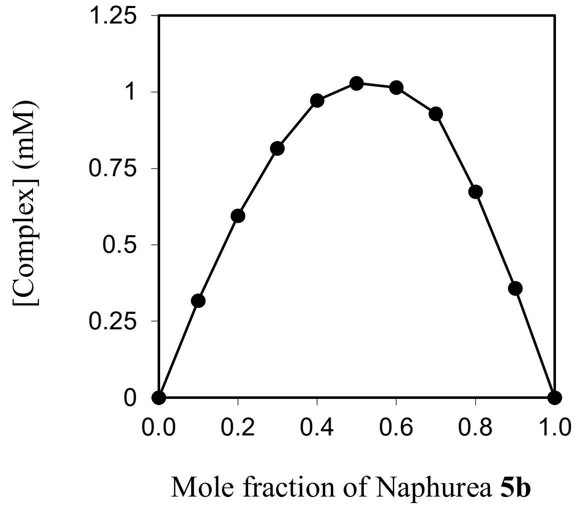

b)

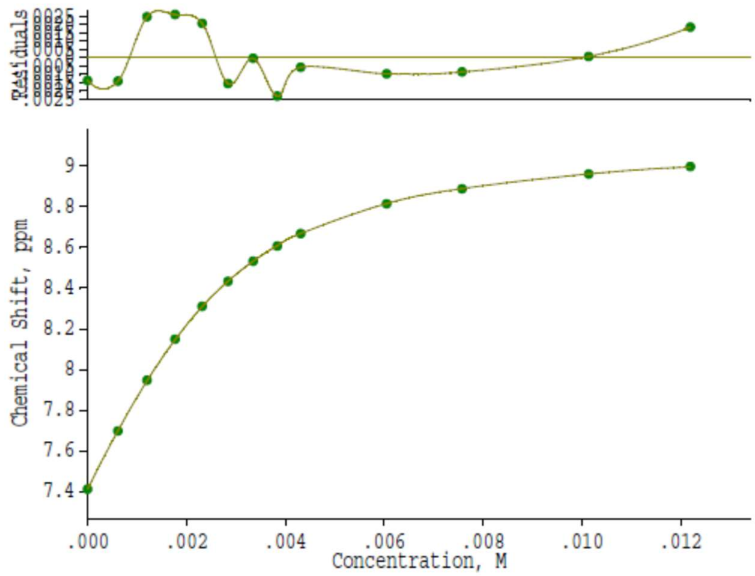

Figure S4. Job plot based on ^{1}H NMR data for (a) Naphurea **5b** + Cl^{-}; (b) Titration data fitted with 1:1 model and residual distribution. Total concentration 2.5×10^{-3} M in $CDCl_3$.

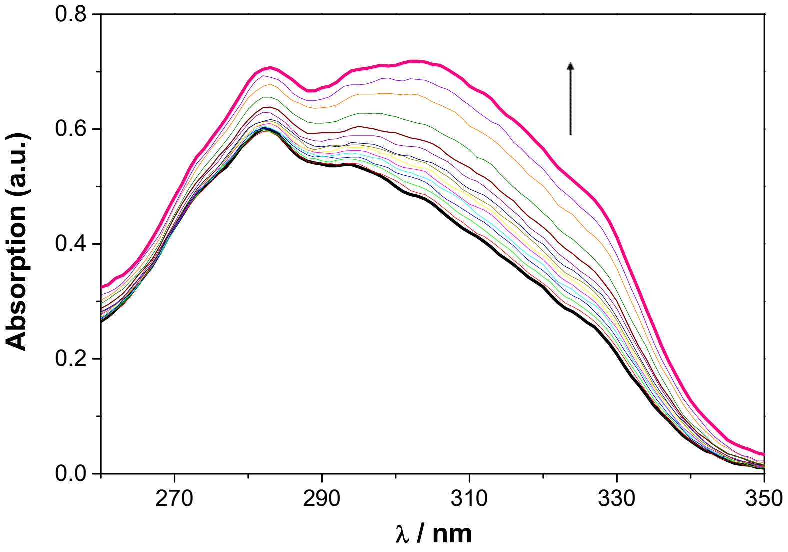

Figure S5. Changes in the absorption spectra of Naph-urea **5b** (3.0×10^{-5} M) upon addition of TBA Cl (up to 10 equiv.) in CH_2Cl_2. The arrow indicates the increasing amounts of salt.

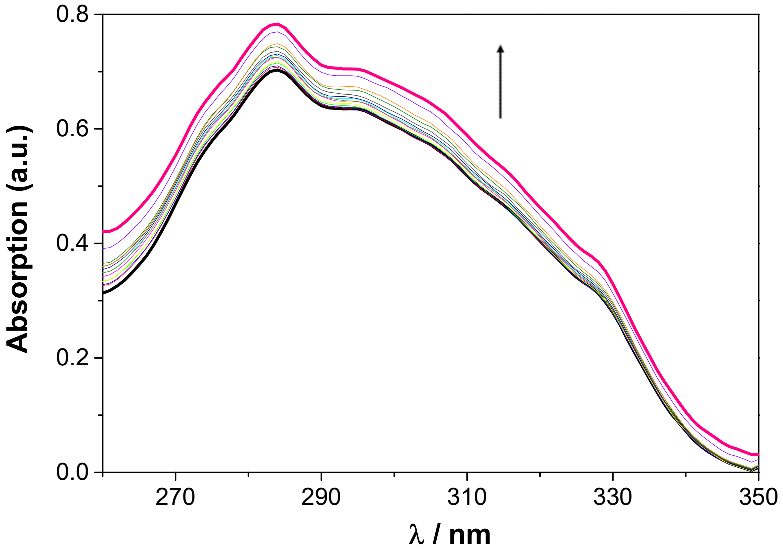

Figure S6. Changes in the absorption spectra of Naph-urea **5a** (5.0×10^{-5} M) upon addition of TBA HSO_4 (up to 10 equiv.) in CH_2Cl_2. The arrow indicates the increasing amounts of salt.

a)

b)

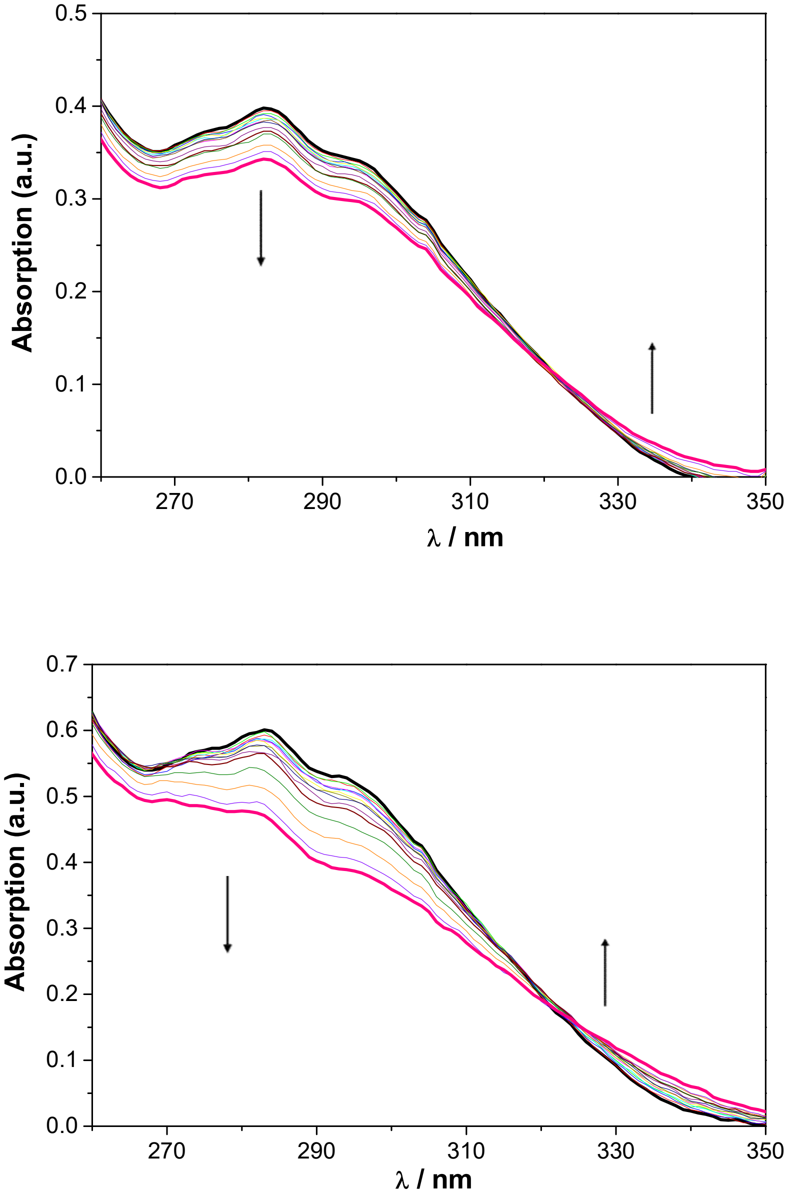

Figure S7. Changes in the absorption spectra of Naph-thiourea **5c** (2.5 × 10^{-5} M) upon addition of: (a) TBA Br (up to 10 equiv) and (b) TBA AcO (up to 10 equiv), in CH_2Cl_2. The arrows indicate the decreasing or increasing amounts of salt.

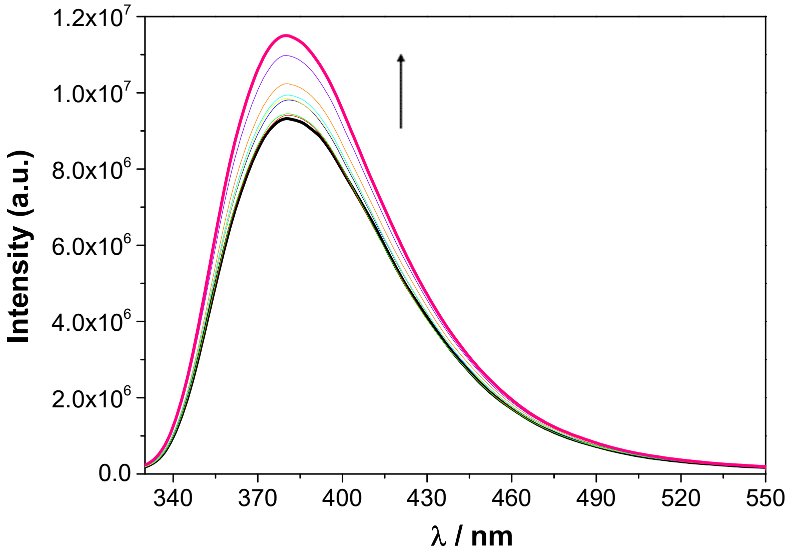

Figure S8. Changes in the emission spectra of Naph-urea **5a** (5.0×10^{-5} M) upon addition of TBA HSO_4 (up to 10 equiv.) in CH_2Cl_2. The arrow indicates the increasing amounts of salt.

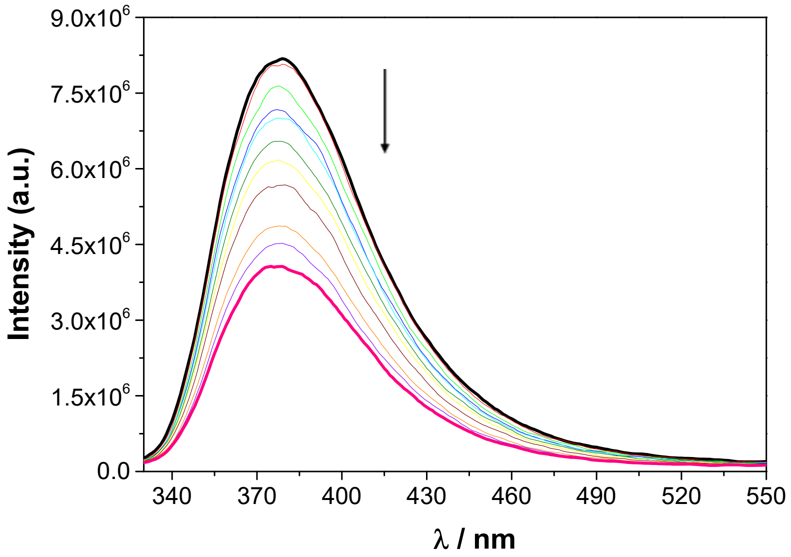

Figure S9. Changes in the emission spectra of Naph-urea **5b** (3.0×10^{-5} M) upon addition of TBA BzO (up to 10 equiv.) in CH_2Cl_2. The arrow indicates the decreasing amounts of salt.

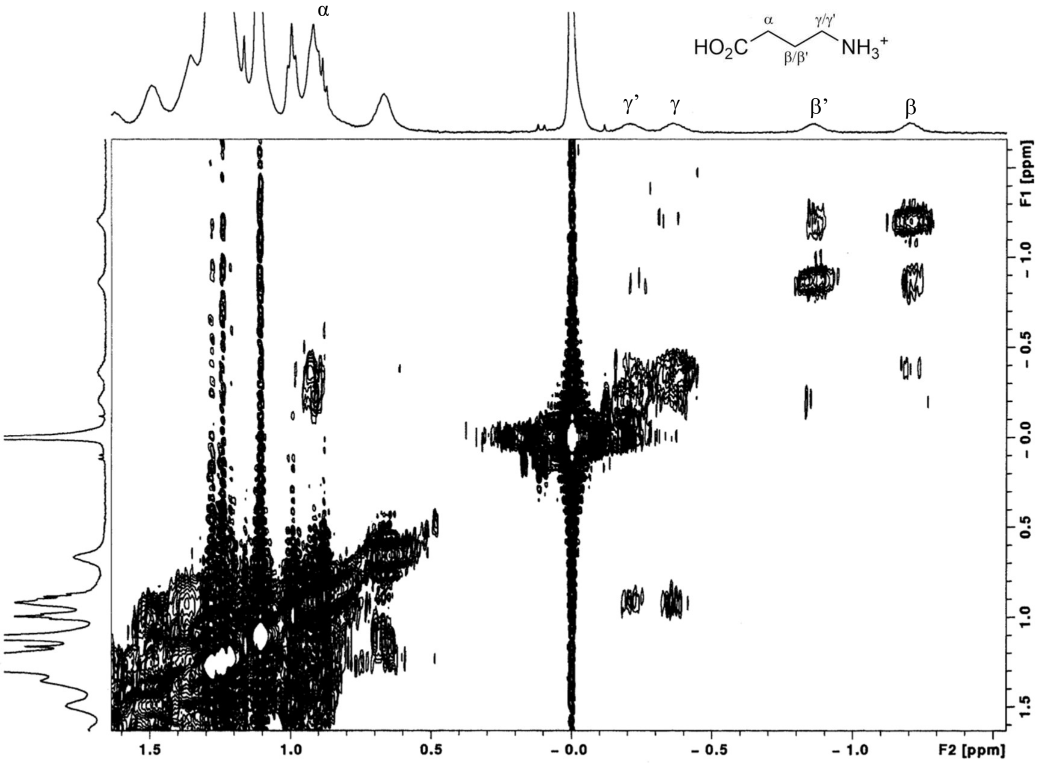

Figure S10. Section of the COSY spectrum (500 MHz, 233 K, $CDCl_3$/MeOD, 5:1, v/v) of [**5a**] = [GABA·HCl] = 1.0 mM.

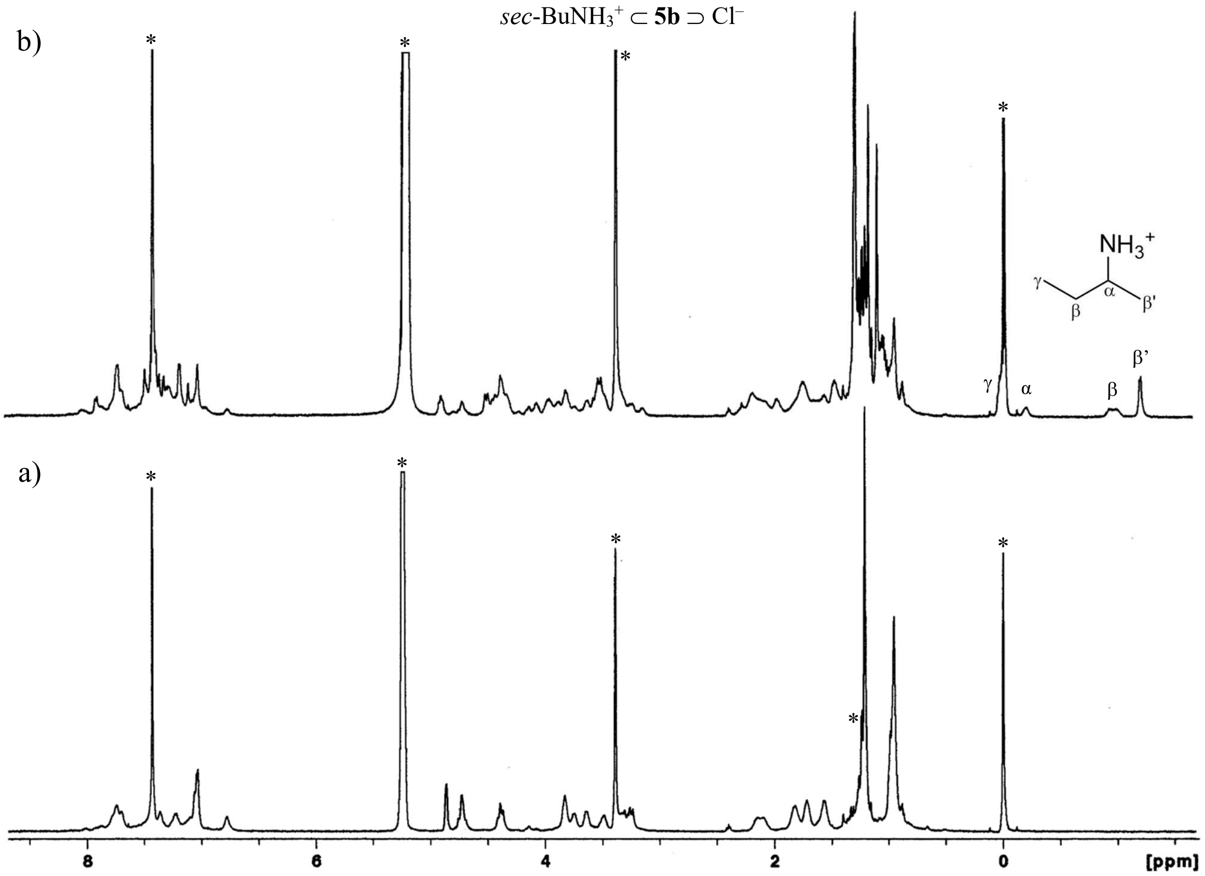

Figure S11. ^{1}H NMR spectra (500 MHz, 223 K, $CDCl_3/CD_3OD$, 5:1, v/v) of: a) [**5b**] = 1.0 mM; b) [**5b**] = [*sec*-$BuNH_2$·HCl] = 1 mM. *Denotes residual solvent signals.

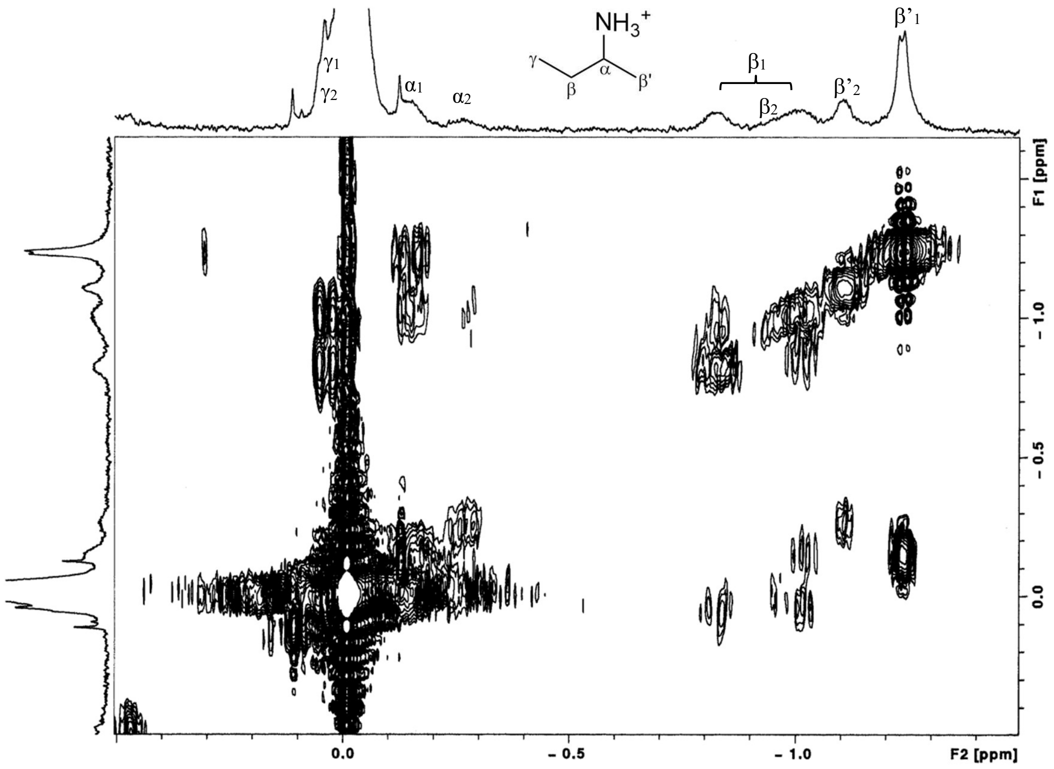

Figure S12. Section of the COSY spectrum (500 MHz, 223 K, $CDCl_3$/MeOD, 5:1, v/v) of [**5a**] = [*sec*-$BuNH_2 \cdot HCl$] = 1.0 mM. 1 and 2 mean the two sets of signals for the *s*-Bu group of the guest inside the cavity for the two diastereotopic complexes formed.

	**5a**	**5b**
free X-Ray	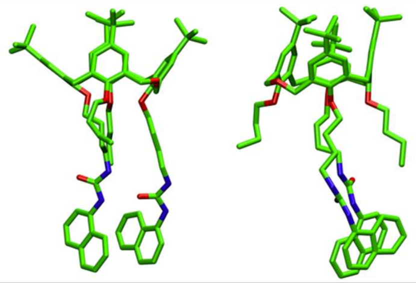	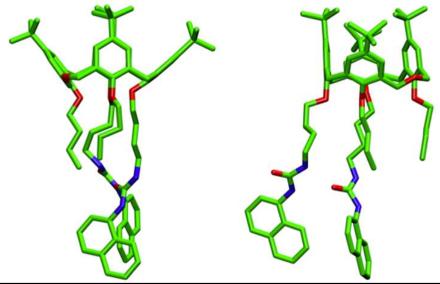
$F^- \subset$Calix	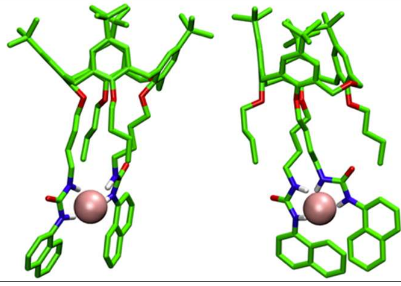	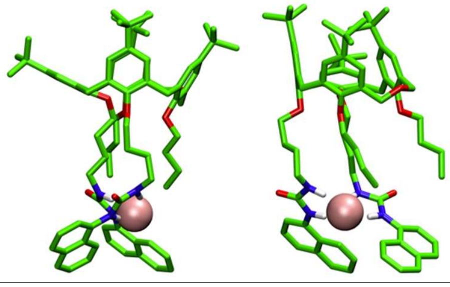

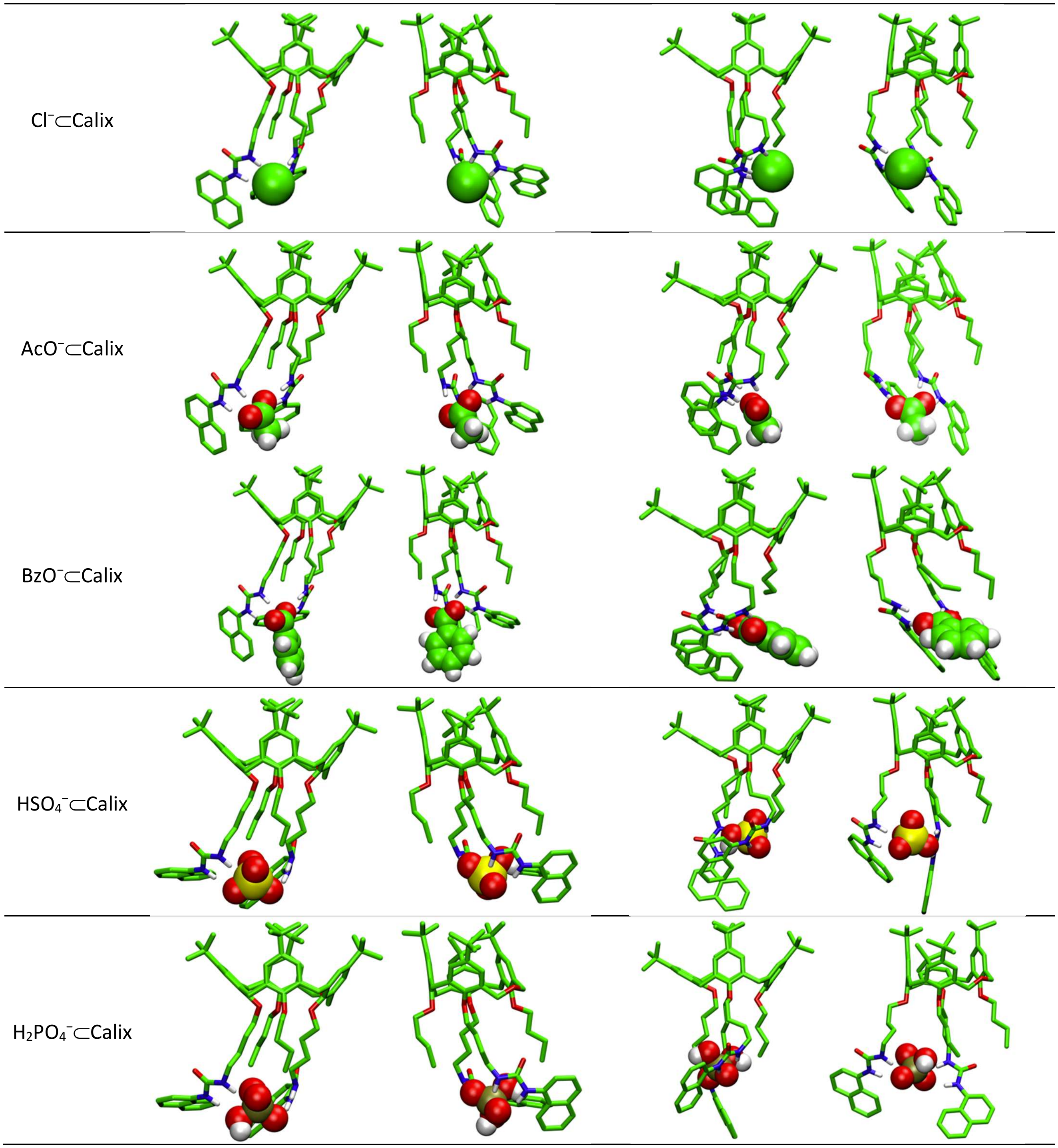

Figure S13. Structures of the free calixarenes (starting point X-Ray structures) and of the $X^{-}\subset$Naphurea complexes after QM optimization (orthogonal views).

Table S3. B3LYP/6-31G(d.p)+BG3BJ total (in hartrees) and complexation energies ΔE (in kJ.mol^{-1}) for the complexed calixarenes

	E (hartrees)			ΔE (kJ.mol^{-1})	
Host	**5a**	**5b**	ions	**5a**	**5b**
free RX	−3973.0223	−3973.0217			
F^-	−4072.9665	−4072.9881	−99.7540	−498.9	−557.1
Cl^-	−4433.3517	−4433.3574	−460.2522	−202.5	−219.0
AcO^-	−4201.6303	−4201.6411	−228.5083	−261.5	−291.4
BzO^-	−4393.3982	−4393.4171	−420.2874	−232.1	−283.3
HSO_4^-	−4672.7955	−4672.8002	−699.6999	−192.3	−206.2
$H_2PO_4^-$	−4616.7133	−4616.7188	−643.6069	−220.6	−236.6
n-$PrNH_3^+Cl^-$	−4608.4150	−4608.4431	−635.1120	−736.3	−811.6
n-$BuNH_3^+Cl^-$	−4647.7331	−4647.7527	−674.4605	−656.5	−709.5
(*R*)-*sec*-$BuNH_3^+$ Cl^- / (*M*)-**5a**	−4647.7585		−674.4678	−704.1	
(*S*)-*sec*-$BuNH_3^+$·Cl^- / (*M*)-**5a**	−4647.7616		−674.4678	−712.4	
(*R*)-*sec*-$BuNH_3^+$ Cl^- / (*P*)-**5a**	−4647.7535		−674.4678	−691.1	
(*S*)-*sec*-$BuNH_3^+$·Cl^- / (*P*)-**5a**	−4647.7551		−674.4678	−695.3	

Table S4. B3LYP/6-31G(d.p)+BG3BJ optimized H-bond distances (in hartrees)

			Distances (Å)				Averaged Value (Å)
F^-	**5a**	$H_{N\text{-calix}}\cdots F^-$	1.660	1.710	1.804	1.813	1.747
	**5b**	$H_{N\text{-calix}}\cdots F^-$	1.695	1.732	1.758	1.800	1.746
Cl^-	**5a**	$H_{N\text{-calix}}\cdots Cl^-$	2.242	2.270	2.331	2.357	2.300
	**5b**	$H_{N\text{-calix}}\cdots F^-$	2.258	2.284	2.310	2.498	2.338
AcO^-	**5a**	$H_{N\text{-calix}}\cdots O_{AcO}$	1.817	1.876	1.897	1.935	1.881
	**5b**	$H_{N\text{-calix}}\cdots O_{AcO}$	1.839	1.851	1.916	1.982	1.897
BzO^-	**5a**	$H_{N\text{-calix}}\cdots O_{BzO}$	1.809	1.875	1.923	1.961	1.892
	**5b**	$H_{N\text{-calix}}\cdots O_{BzO}$	1.853	1.884	1.944	1.966	1.912
HSO_4^-	**5a**	$H_{N\text{-calix}}\cdots O_{HSO}$	1.973	2.018	2.046	2.045	2.021
	**5b**	$H_{N\text{-calix}}\cdots O_{HSO}$	1.963	1.970	2.039	2.094	2.017
$H_2PO_4^-$	**5a**	$H_{N\text{-calix}}\cdots O_{HPO}$	1.866	1.939	1.995	2.035	1.959
	**5b**	$H_{N\text{-calix}}\cdots O_{HPO}$	1.963	1.970	2.039	2.094	2.017
n-$PrNH_3^+\cdot Cl^-$	**5a**	$H_{N\text{-calix}}\cdots Cl^-$	2.202	2.274	2.320	2.406	2.308
	**5a**	$O_{calix}\cdots H_N$	1.778	1.983	2.684		
	**5b**	$H_{N\text{-calix}}\cdots Cl^-$	2.207	2.305	2.413	2.439	2.341
	**5b**	$O_{calix}\cdots H_N$	1.671	1.825	2.355		
n-$BuNH_3^+\cdot Cl^-$	**5a**	$H_{N\text{-calix}}\cdots Cl^-$	2.215	2.221	2.276	2.422	2.284
	**5a**	$O_{calix}\cdots H_N$	1.944	2.294	2.887		
	**5b**	$H_{N\text{-calix}}\cdots Cl^-$	2.280	2.280	2.326	2.406	2.323
	**5b**	$O_{calix}\cdots H_N$	1.650	1.802	2.438		2.434
(*R*)-*s*-$BuNH_3^+\cdot Cl^-$	(*M*)-**5a**	$H_{N\text{-calix}}\cdots Cl^-$	2.159	2.218	2.421	2.460	2.314
	(*M*)-**5a**	$O_{calix}\cdots H_N$	1.788	1.841	2.596		
(*S*)-*s*-$BuNH_3^+\cdot Cl^-$	(*M*)-**5a**	$H_{N\text{-calix}}\cdots Cl^-$	2.158	2.221	2.418	2.466	2.316
	(*M*)-**5a**	$O_{calix}\cdots H_N$	1.783	1.870	3.207		
(*R*)-*s*-$BuNH_3^+\cdot Cl^-$	(*P*)-**5a**	$H_{N\text{-calix}}\cdots Cl^-$	2.193	2.214	2.355	2.529	2.322
	(*P*)-**5a**	$O_{calix}\cdots H_N$	1.759	1.808	3.213		
(*S*)-*s*-$BuNH_3^+\cdot Cl^-$	(*P*)-**5a**	$H_{N\text{-calix}}\cdots Cl^-$	2.193	2.212	2.347	2.532	2.321
	(*P*)-**5a**	$O_{calix}\cdots H_N$	1.761	1.847	3.178		

Table S5. B3LYP/6-31G(d.p)+BG3BJ deformation energies $\Delta_{def}E$ (in $kJ.mol^{-1}$) of the calixarenes (E (complexed calixarene)-E (free calixarene)).

	**5a**	**5b**
F^-	143.0	94.4
Cl^-	111.2	93.4
AcO^-	118.6	72.9
BzO^-	113.3	74.8
HSO_4^-	116.7	129.8
$H_2PO_4^-$	118.6	129.8

X-ray structure refinement details

The asymmmetric units of the monoclinic crystals of **5a** and **5b** (both in P21/n space group) each contain one dihomooxacalix[4]arene molecule, with no co-crystallised solvent molecules. Two-position disorder was found in various parts of both molecules. In the case of **5a**, the molecule shows a disorder of one naphthalene ring (ring **A**), as well as the nitrogen atom to which the ring is bonded. These were refined at 0.50/0.50 partial occupancies. In addition, one of the butoxy rings shows disorder for all four of the carbon atoms, which were refined at 0.85/0.15 partial occupancies. A SIMU restraint was applied to the two nearly overlapped parts of the first atom of the disordered chain. In the case of **5b**, a similar disorder was observed for one of the butoxy chains, for which the final refinement was performed at 0.75/0.25 partial occupancies.

Table S6. Crystal data and structure refinement for **5a** and **5b**

	**5a**	**5b**
Empirical formula	C_{83} H_{106} N_4 O_7	C_{83} H_{106} N_4 O_7
Formula weight	1271.71	1271.71
Temperature (K)	100(2)	100(2)
Wavelength (Å)	0.7	0.7
Crystal system	Monoclinic	Monoclinic
Space group	$P\ 2_1$/n	$P\ 2_1$/n
Unit cell dimensions (Å, °)	a = 17.016(11), α = 90	a = 11.473(9), α = 90
	b = 16.638(5), β = 102.24(3)	b = 55.43(3), β = 102,06(8)
	c = 27.049(9), γ = 90	c = 12.189(10), γ = 90
Volume (Å^3)	7484(6)	7581(10)
Z	4	4
ρ_{calcd} (g/cm^3)	1,129	1.114
μ (mm^{-1})	0.068	0.069
F(000)	2752	2752
Reflections collected	42495	35512
Independent reflections	10696	6969
Data / restraints / parameters	10696 / 6 / 904	6969 / 0 /878
GooF	1.027	1.03
Final R indices [I>2σ(I)]	R_1 = 0.0926	R_1 = 0.0826
	wR_2 = 0.2555	wR_2 = 0.2148
R indices (all data)	R_1 = 0.1355	R_1 = 0.1418
	wR_2 = 0.2944	wR_2 = 0.2625
CCDC code	2015473	2015474

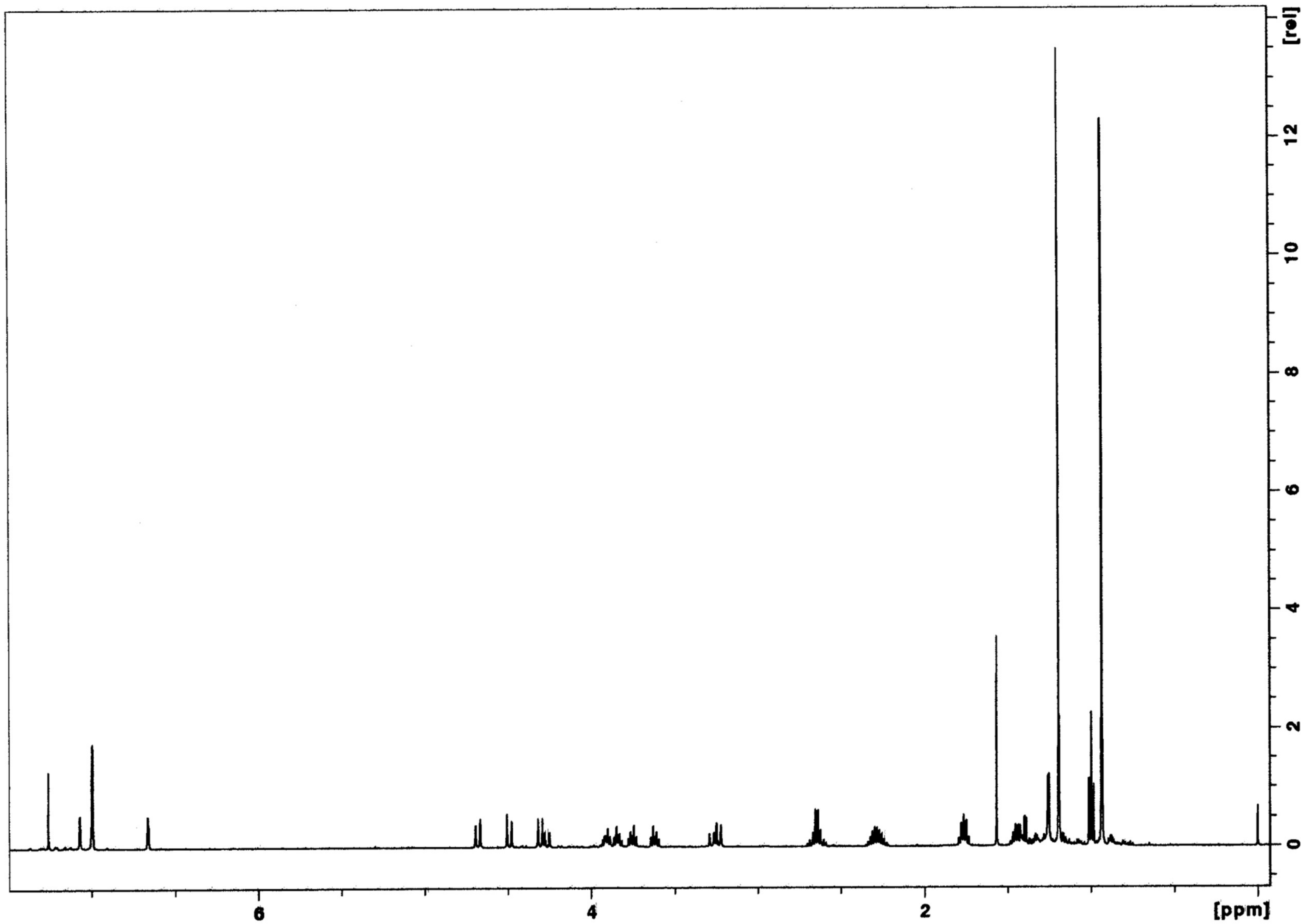

Figure S14. ^{1}H NMR spectrum (500 MHz, $CDCl_3$, rt) of bis(cyanopropyl) **3b**.

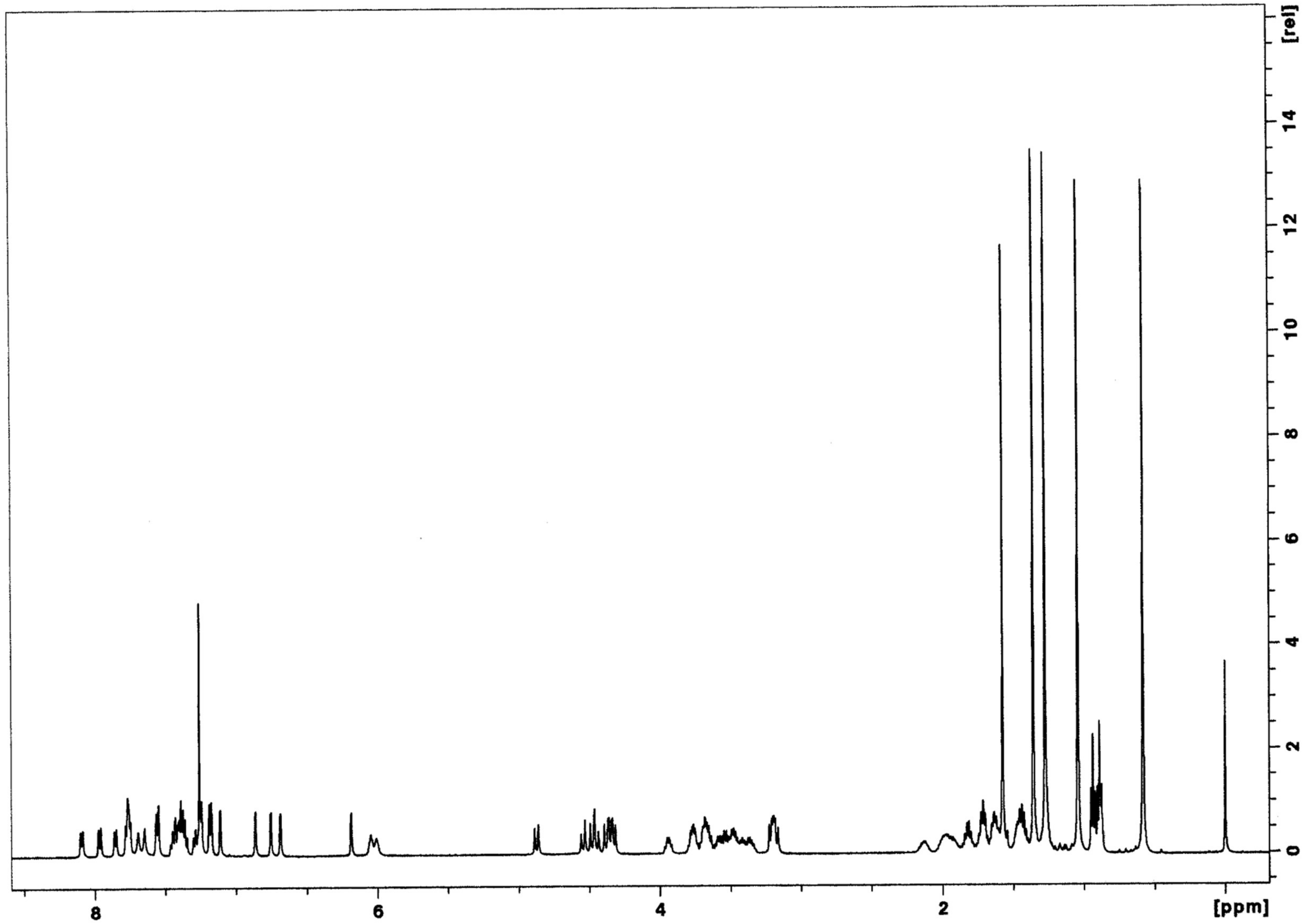

Figure S15. ^{1}H NMR spectrum (500 MHz, $CDCl_3$, rt) of asymmetric Naphurea **5a**.

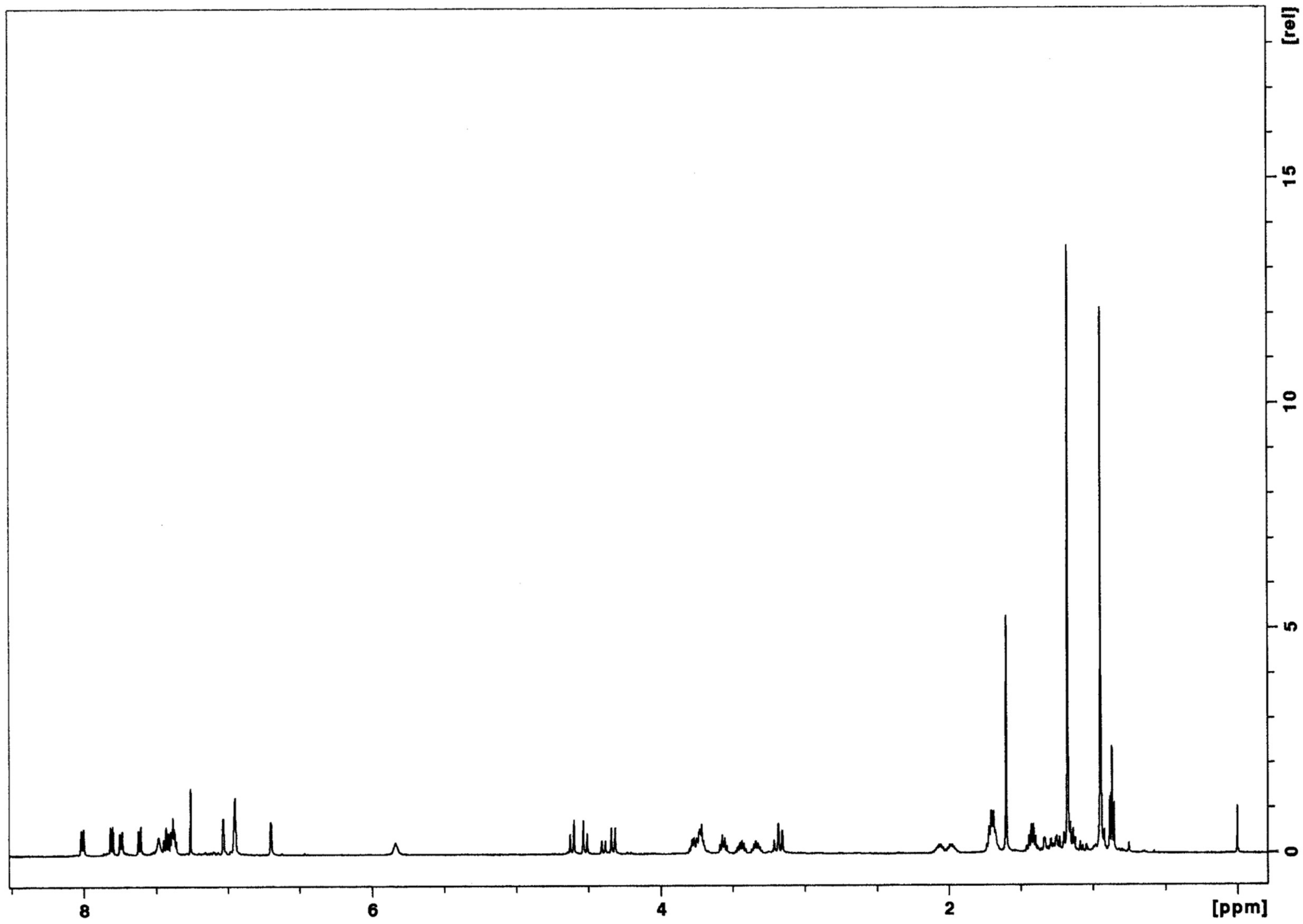

Figure S16. ^{1}H NMR spectrum (500 MHz, $CDCl_3$, rt) of symmetric Naphurea **5b**.

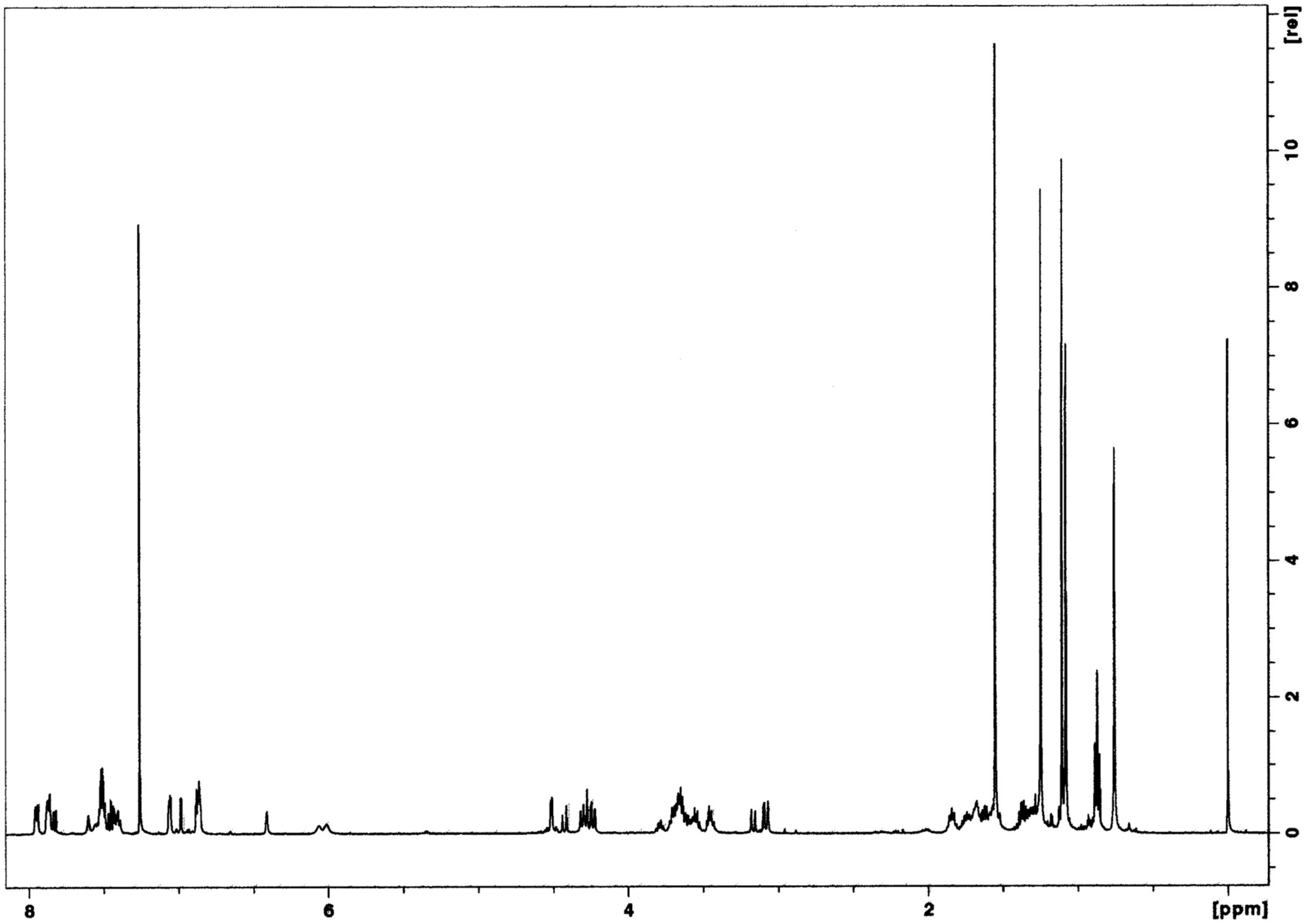

Figure S17. ^{1}H NMR spectrum (500 MHz, $CDCl_3$, rt) of Naphthiourea **5c**.

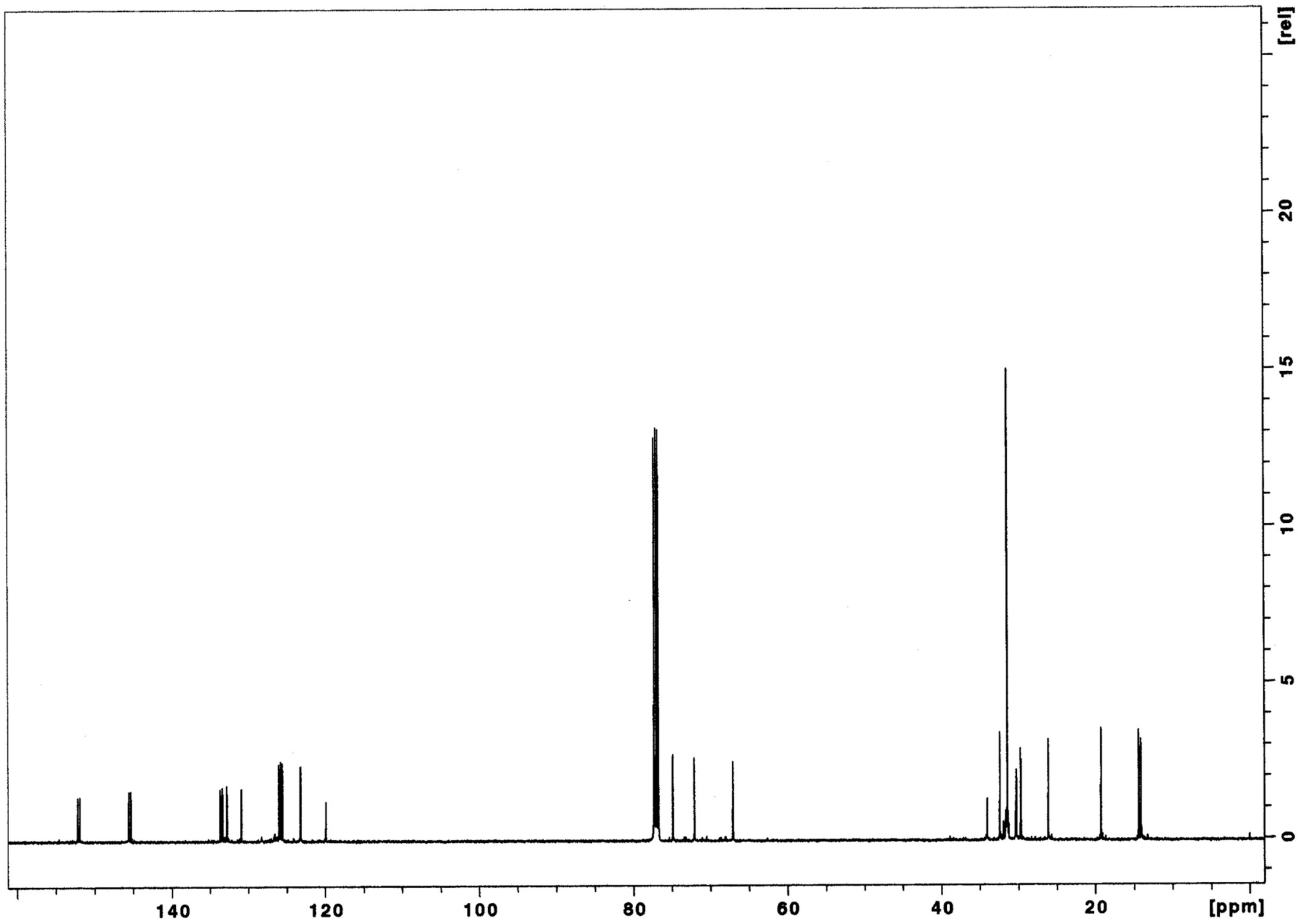

Figure S18. ^{13}C NMR spectrum (125.8 MHz, $CDCl_3$, rt) of bis(cyanopropyl) **3b**.

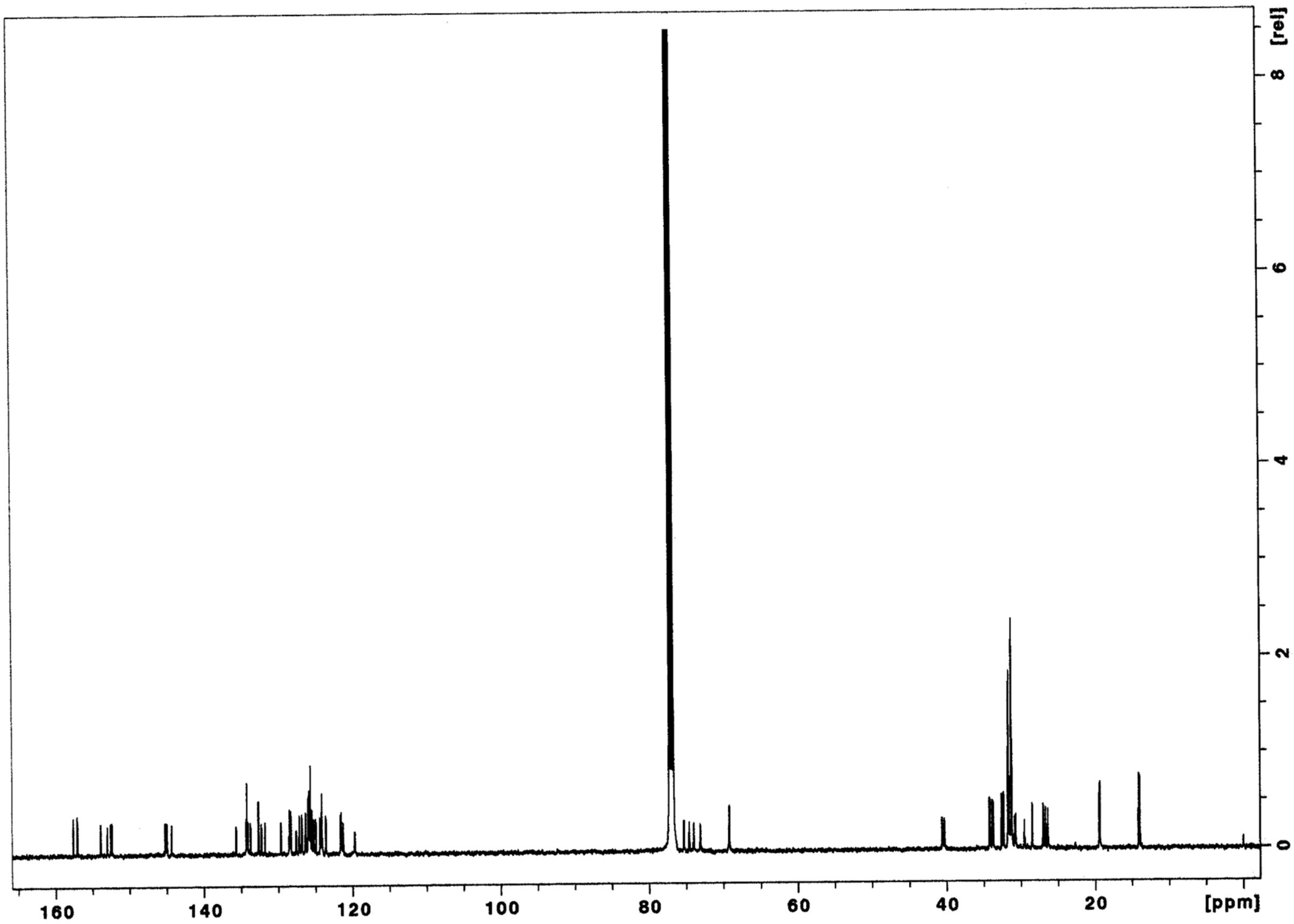

Figure S19. ^{13}C NMR spectrum (125.8 MHz, $CDCl_3$, rt) of asymmetric Naphurea **5a**.

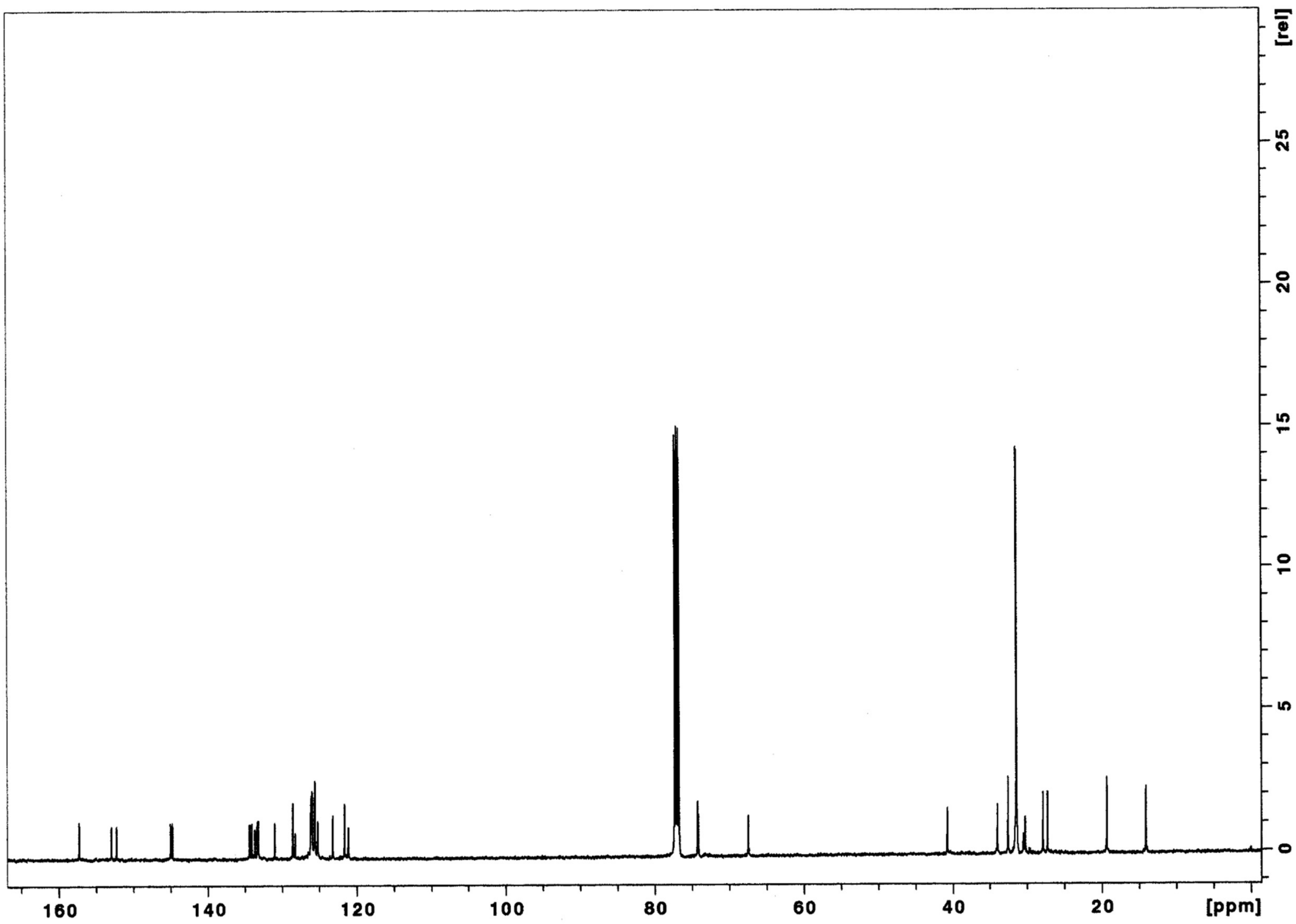

Figure S20. ^{13}C NMR spectrum (125.8 MHz, $CDCl_3$, rt) of symmetric Naphurea **5b**.

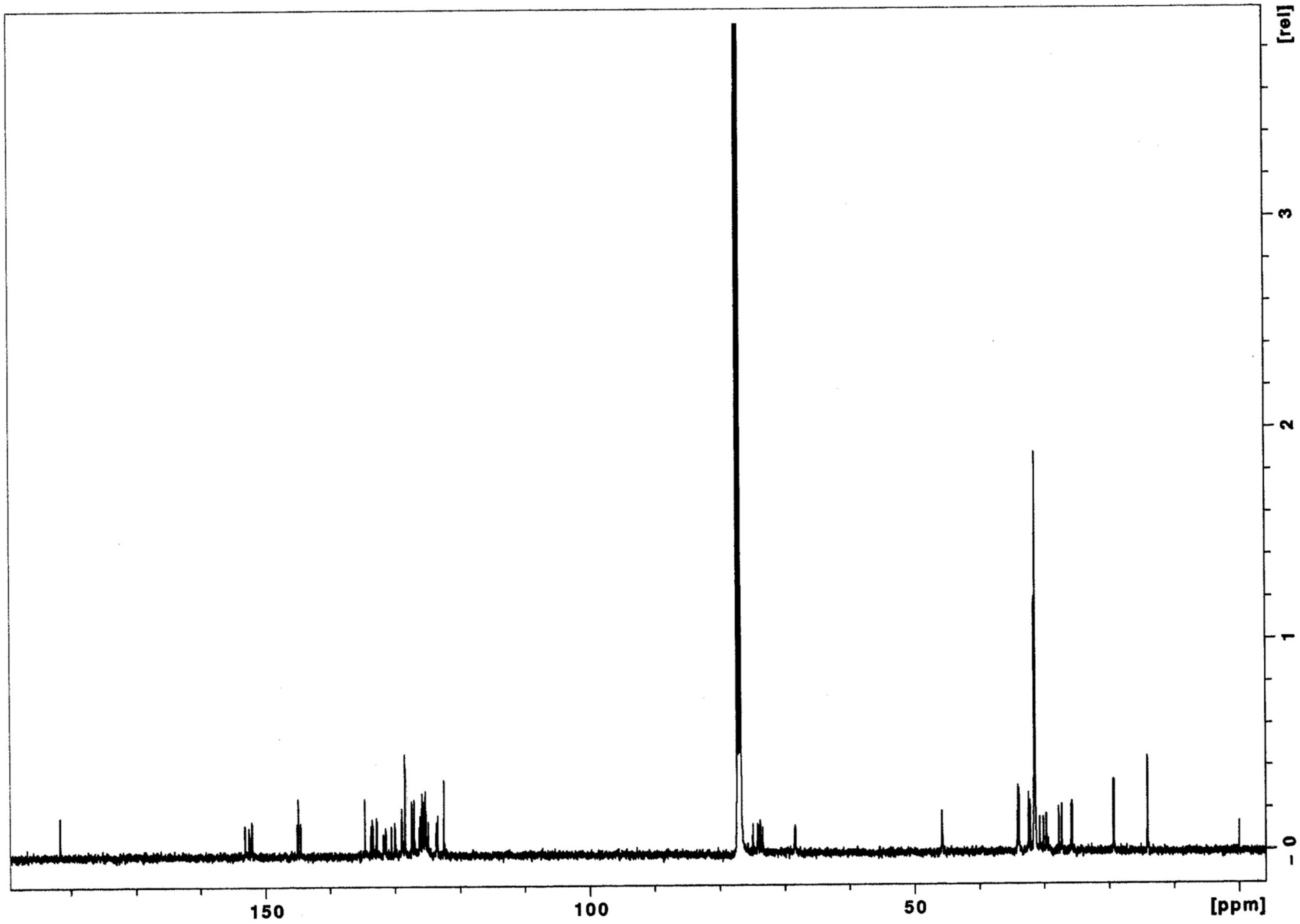

Figure S21. ^{13}C NMR spectrum (125.8 MHz, $CDCl_3$, rt) of Naphthiourea **5c**.

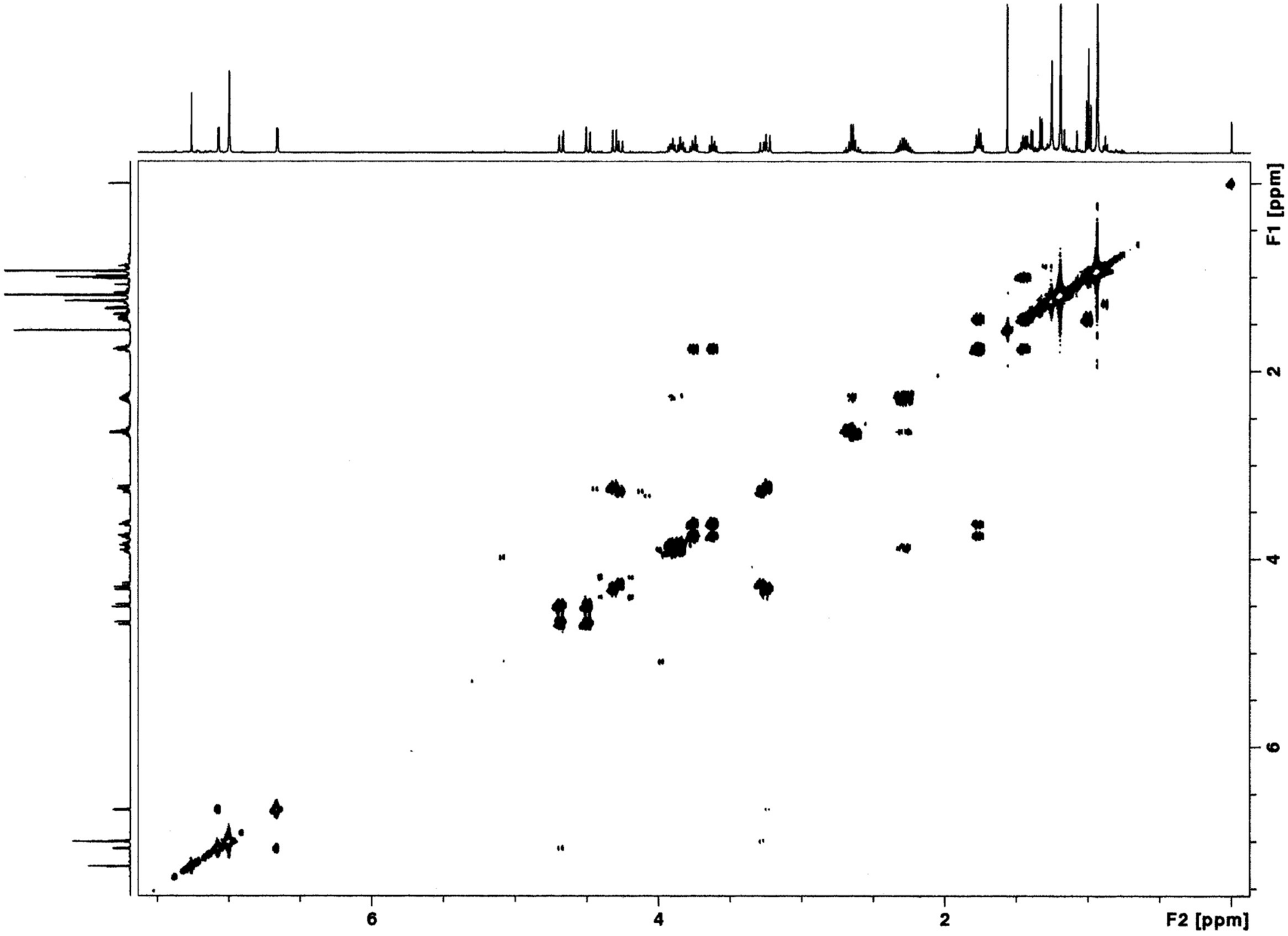

Figure S22. COSY spectrum (500 MHz, $CDCl_3$, rt) of bis(cyanopropyl) **3b**.

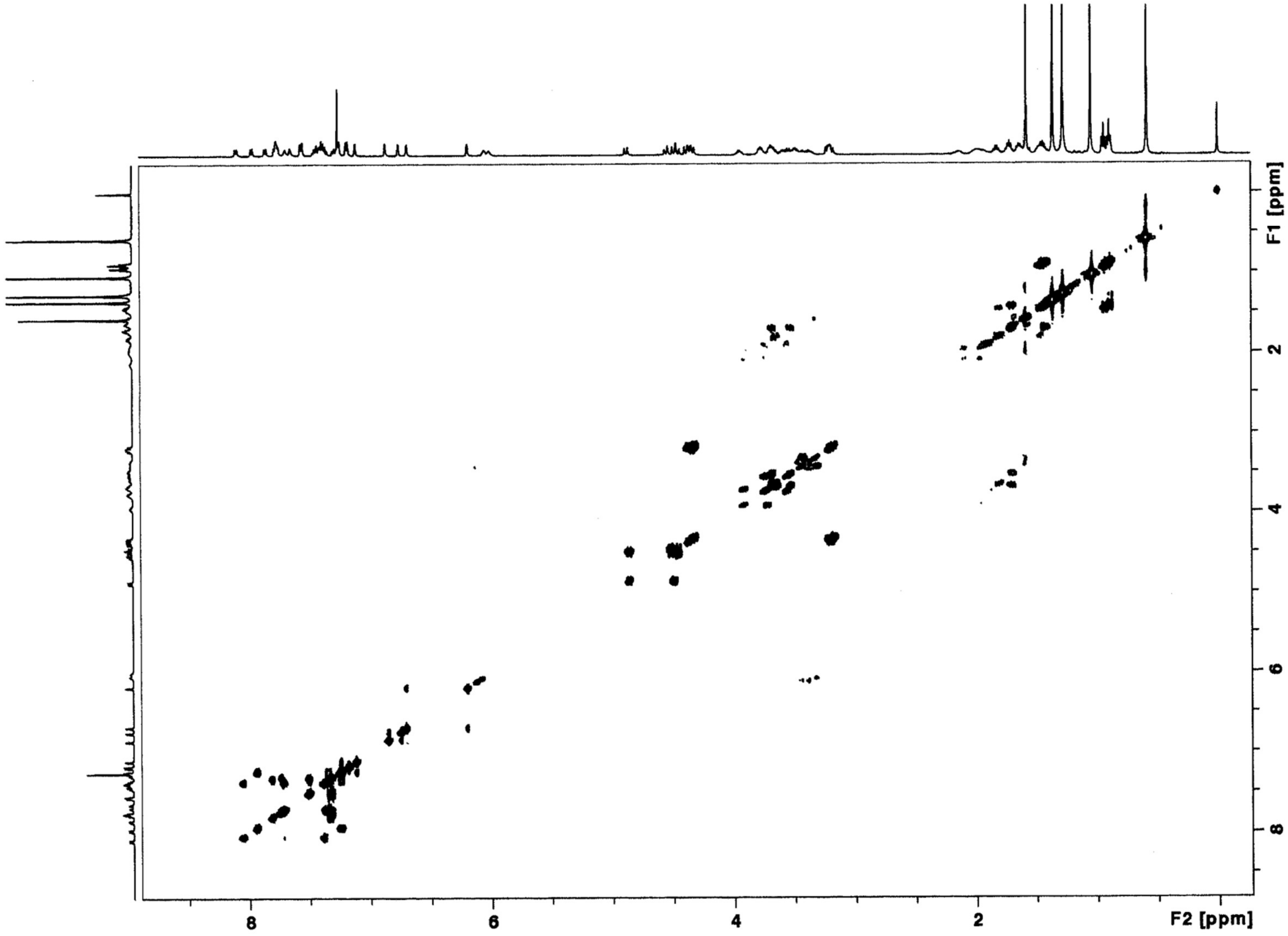

Figure S23. COSY spectrum (500 MHz, $CDCl_3$, rt) of asymmetric Naphurea **5a**.

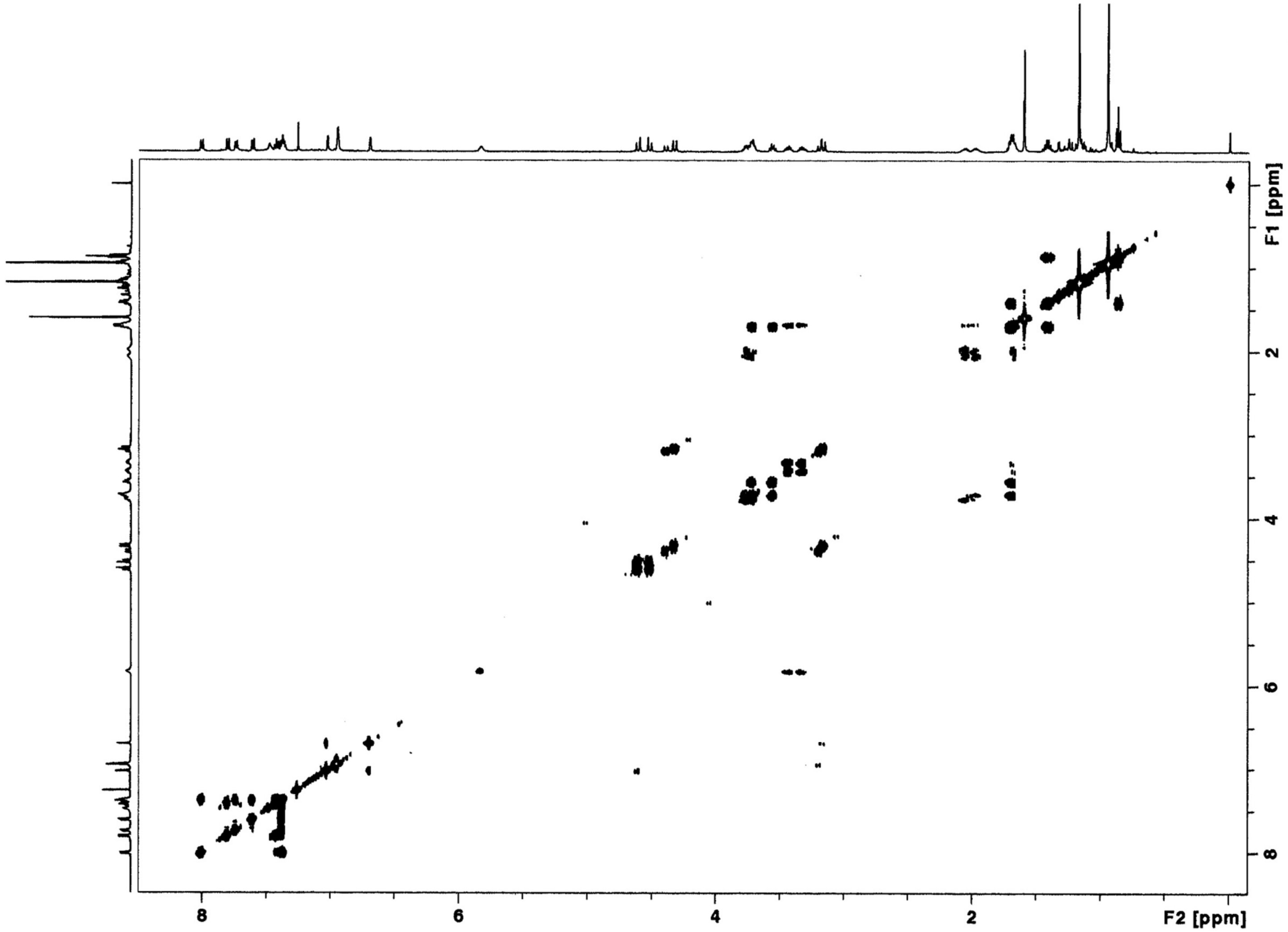

Figure S24. COSY spectrum (500 MHz, $CDCl_3$, rt) of symmetric Naphurea **5b**.

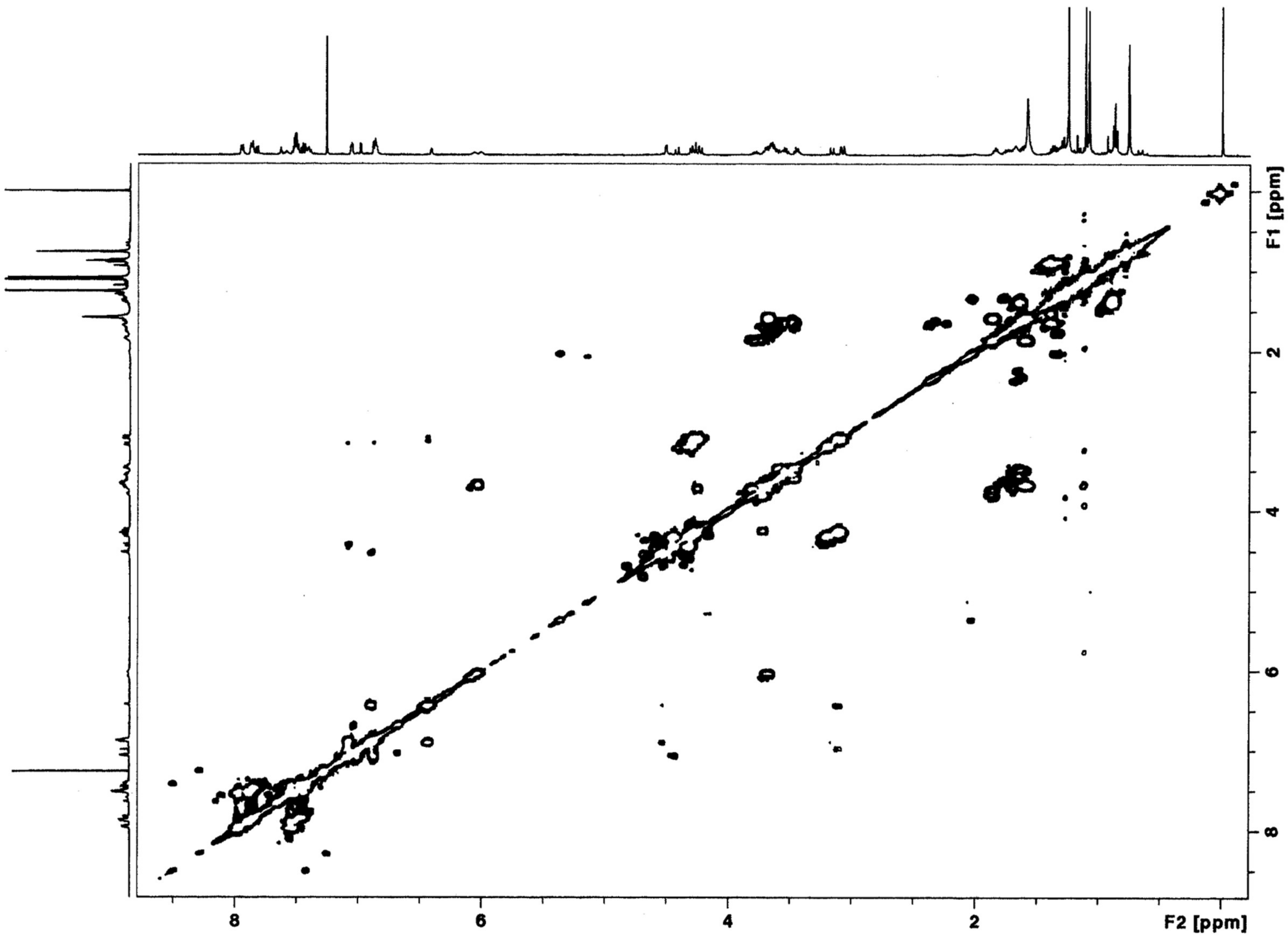

Figure S25. COSY spectrum (500 MHz, $CDCl_3$, rt) of of Naphthiourea **5c**.
